# Microbial β-glucuronidases drive human periodontal disease etiology

**DOI:** 10.1126/sciadv.adg3390

**Published:** 2023-05-05

**Authors:** Adam D. Lietzan, Joshua B. Simpson, William G. Walton, Parth B. Jariwala, Yongmei Xu, Marcella H. Boynton, Jian Liu, Matthew R. Redinbo

**Affiliations:** ^1^ Division of Oral and Craniofacial Health Sciences, Adams School of Dentistry, University of North Carolina at Chapel Hill, Chapel Hill, NC 27599, USA.; ^2^ Department of Chemistry, University of North Carolina at Chapel Hill, Chapel Hill, NC 27599, USA.; ^3^ Department of Chemical Biology and Medicinal Chemistry, University of North Carolina at Chapel Hill, Chapel Hill, NC 27599, USA.; ^4^ Division of General Medicine and Clinical Epidemiology, University of North Carolina at Chapel Hill, Chapel Hill, NC 27599, USA.; ^5^ North Carolina Translational and Clinical Sciences Institute, University of North Carolina at Chapel Hill, Chapel Hill, NC 27599, USA.; ^6^ Integrated Program for Biological and Genome Sciences, University of North Carolina at Chapel Hill, Chapel Hill, NC 27599, USA.; ^7^ Department of Biochemistry and Biophysics, University of North Carolina at Chapel Hill, Chapel Hill, NC 27599, USA.; ^8^ Department of Microbiology and Immunology, University of North Carolina at Chapel Hill, Chapel Hill, NC 27599, USA.

## Abstract

Periodontitis is a chronic inflammatory disease associated with persistent oral microbial dysbiosis. The human β-glucuronidase (GUS) degrades constituents of the periodontium and is used as a biomarker for periodontitis severity. However, the human microbiome also encodes GUS enzymes, and the role of these factors in periodontal disease is poorly understood. Here, we define the 53 unique GUSs in the human oral microbiome and examine diverse GUS orthologs from periodontitis-associated pathogens. Oral bacterial GUS enzymes are more efficient polysaccharide degraders and processers of biomarker substrates than the human enzyme, particularly at pHs associated with disease progression. Using a microbial GUS-selective inhibitor, we show that GUS activity is reduced in clinical samples obtained from individuals with untreated periodontitis and that the degree of inhibition correlates with disease severity. Together, these results establish oral GUS activity as a biomarker that captures both host and microbial contributions to periodontitis, facilitating more efficient clinical monitoring and treatment paradigms for this common inflammatory disease.

## Teaser

Elevated β-glucuronidase activity tracks with pathogenic oral microbes in gum disease.

## INTRODUCTION

Chronic inflammation is a notable global health burden, with more than 50% of all deaths attributable to inflammation-related diseases (*1*). Periodontitis (gum disease) is the sixth most prevalent health condition in the world and affects 42% of dentate adults in the United States (*2*, *3*). It is a microbially associated chronic inflammatory condition that results in the destruction of periodontal tissues and tooth loss. Local and systemic cellular mechanisms link periodontal disease to inflammatory comorbidities (*4*). Persistent exposure to pathogenic microbial taxa in untreated periodontitis produces chronic inflammation that worsens systemic disorders, such as cardiovascular, gastrointestinal (GI), and neurodegenerative diseases, and diabetes (*5*). Personalized periodontal medicine seeks to integrate genomic information, environmental factors, and diagnostic testing to guide precision treatments for both oral and overall health (*6*). Thus, point-of-care biomarkers fully reflective of disease etiology and progression are critical to addressing the local and systemic burden of periodontitis.

As periodontal inflammation develops, host immune cells migrate to the oral mucosal barrier and, upon stimulation by a dysbiotic oral microbiota, degranulate and/or undergo apoptosis to release their intracellular contents (*7*). Specifically, polymorphonuclear leukocytes (neutrophils) transmigrate into the periodontal sulcus and contribute their contents to the gingival crevicular fluid (GCF) (*8*). One human enzyme released from activated neutrophils is β-glucuronidase (GUS), and its activity is a measure of neutrophil degranulation (*9*, *10*). Human GUS is an exoglycosidase that hydrolyzes the terminal glucuronic acid (GlcA) from the nonreducing end of polysaccharides and reporter substrates (*11*). In health, GUS is necessary for GlcA-containing glycosaminoglycan turnover and extracellular matrix homeostasis. In periodontitis, however, GUS contributes to extracellular matrix degradation and periodontal inflammation. GUS activity from GCF or saliva has been used as an indicator of periodontal disease development since the 1980s (*12*, *13*).

It has long been assumed that the GUS activity used as a biomarker for periodontitis arose solely from the human enzyme. However, recent reports have demonstrated that the human GI microbiome contains an assembly of unique microbial GUS enzymes with variable enzymatic activities, including the processing of reporter substrates and polysaccharides (*14*–*22*). Given that microbial stimulation is key to the onset and advancement of periodontal inflammation, we hypothesized that nonhuman GUS enzymes contribute to this biomarker for periodontitis. This hypothesis challenges the dogma that GUS activity from oral clinical samples is derived only from host immune cells but, if validated, would expand our understanding of periodontal disease etiology.

Here, we generate the first atlas of microbial GUS proteins in the human oral microbiome and show that it consists of 53 unique enzymes. The structural and functional characterization of GUS enzymes from periodontitis-associated genera highlight unique features that influence active site accessibility and diverse activities. We show that oral microbial GUS enzymes are superior to the human enzyme for processing the reporter substrate used as a biomarker for periodontal disease. We also demonstrate that microbial GUS enzymes act on polysaccharides like those found in periodontal connective tissue and are considerably more active than the human protein at pH values relevant to periodontitis. Last, using an inhibitor selective for microbial GUS enzymes, we demonstrate that GUS activity is reduced in clinical samples obtained from individuals with untreated periodontitis and that the degree of inhibition is directly related to periodontitis severity. Thus, our findings establish that microbial enzymes substantially contribute to GUS activity measured in periodontitis and that activity increases as the disease progresses. These results advance our understanding of periodontal inflammation by outlining a microbial component associated with the disease and highlighting a simple diagnostic tool with the potential to stratify individuals at risk for periodontitis. Insights gained from these studies call attention to the potential of microbial GUSs as druggable targets for the treatment of periodontitis.

## RESULTS

### The human oral microbiome contains GUS proteins

The human oral microbiome is part of the >700 microbial species within the human aerodigestive tract (*23*). The expanded and curated Human Oral Microbiome Database (eHOMD; homd.org) contains 2123 oral/nasal genomes and >5 million translated protein sequences representative of the rich genetic diversity present. To identify the genes that encode GUS enzymes from the oral microbiome, a two-step rubric was employed using microbial GUS proteins of known structure and established residues critical for GUS activity (*22*). First, the 5,135,096 translated proteins in the eHOMD (v3) were screened to identify those with ≥25% sequence identities or <0.05 E values with the microbial GUS enzymes of known crystal structures (from *Escherichia coli*, *Bacteroides fragilis*, *Streptococcus agalactiae*, and *Clostridium perfringens*; fig. S1). Second, a pairwise sequence alignment ensured that the active sites contained seven residues essential for GUS activity (*21*, *24*). The outcome of these two steps selected 165 GUS protein sequences from the 774 microbial species in the database. Removing redundant protein sequences sharing ≥90% identity resulted in a final set of 53 unique GUS enzymes that define the oral microbial GUS atlas (data S1).

**Fig. 1.**
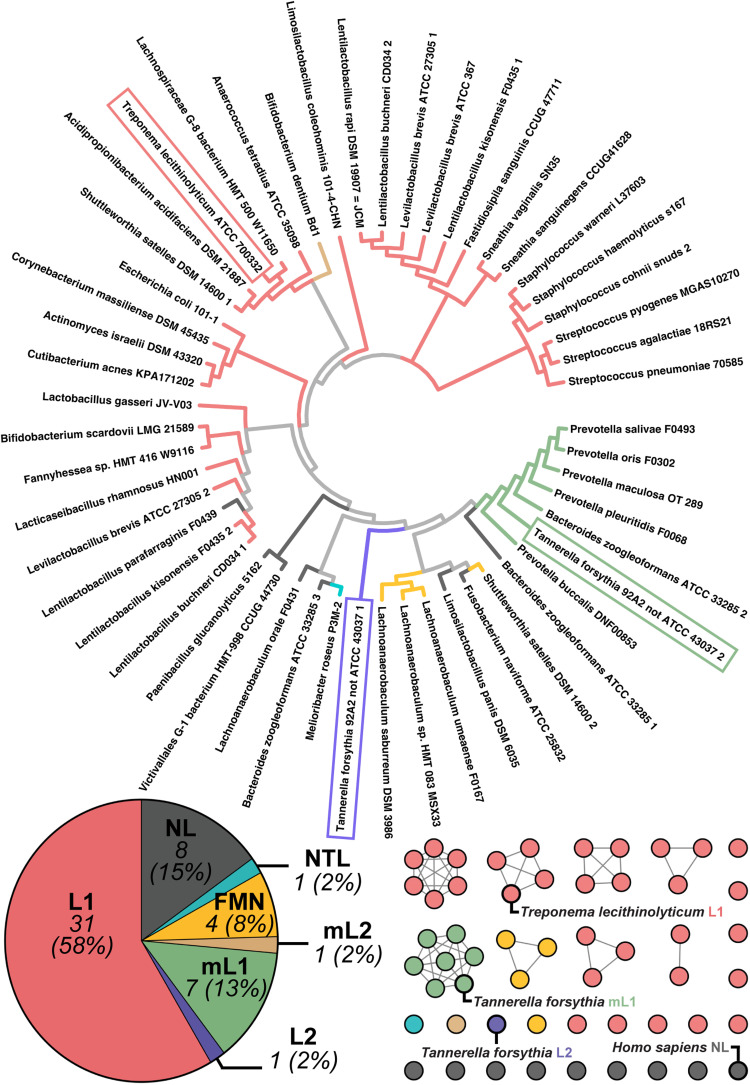
Cladogram and sequence similarity network (SSN) of the nonredundant GUS enzymes from the human oral microbiome with the corresponding loop classifications. A total of 53 nonredundant GUS enzymes were identified from the eHOMD. Highlighted species represent GUS enzymes that were further evaluated via in vitro analysis. Human GUS was included in the SSN but was not identified from the eHOMD.

Although the core glycoside hydrolase family 2 (GH2) fold is conserved among all GUS enzymes of known structure, two loops positioned adjacent to the active site influence substrate binding and catalytic activity. The presence or absence of these loops and their lengths have led to the classification of microbial GUS enzymes into several categories, as outlined previously (*22*): loop 1 (L1), mini-L1 (mL1), loop 2 (L2), mini-loop 2 (mL2), and no loop (NL). Human GUS is classified as an NL enzyme (*22*). In addition, the presence of a unique N-terminal loop (NTL) that contributes amino acids to the active site and a flavin mononucleotide (FMN)–binding site were also used to define two additional structural categories of the oral microbial GUS atlas (*19*, *20*). The 53 sequences in the oral microbial GUS atlas were arranged into a cladogram with branches colored by structural category (Fig. 1). Notably, 58% of the human oral microbial GUS atlas is represented by the L1 structural category (Fig. 1), in sharp contrast to the human gut microbial GUS atlas, in which only 6% are L1 and the majority (55%) are NL (*22*). Furthermore, 42% of the oral L1 enzymes are derived from *Proteobacteria*, also much higher than the 6% observed in the human gut. These data highlight the unique composition of the microbial GUS proteins present in the human oral microbiome compared to that in the human GI tract.

### Oral microbial GUS enzymes have a range of structural diversity

**Fig. 2.**
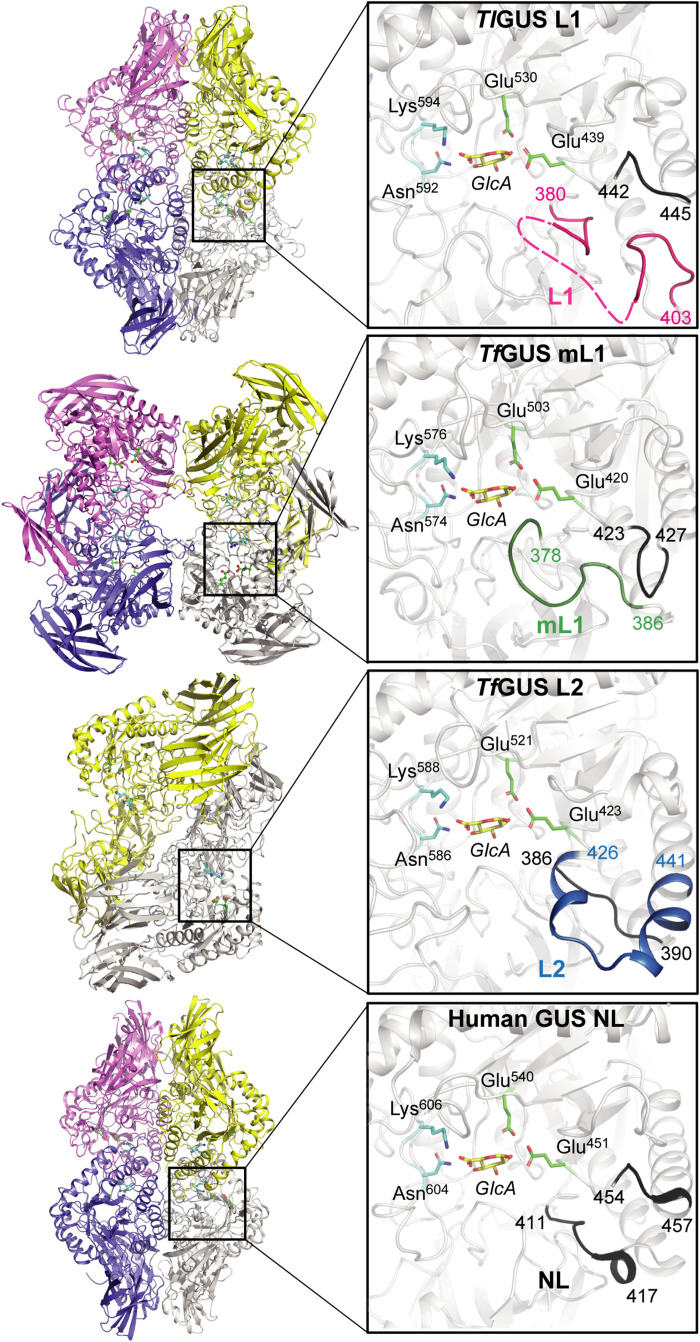
Overall quaternary structure and active site architecture of the selected oral microbial GUS enzymes. The loop category and corresponding loop are highlighted for each enzyme. The crystal structure of the human GUS enzyme is from the previously deposited Protein Data Bank accession code 3HN3. GlcA was modeled into the active site of each enzyme for orientation purposes.

Dysbiosis of the oral microbiota directly affects the progression of periodontitis, with the pathogenic genera *Tannerella*, *Prevotella*, *Treponema*, *Porphyromonas*, and *Fusobacterium* becoming enriched within subgingival plaque (*25*, *26*). To examine the structure and function of unique GUS enzymes from the oral microbiome, we recombinantly expressed and purified the following proteins from bacteria strongly associated with periodontitis (*27*, *28*): L2 and mL1 proteins from *Tannerella forsythia* (*Tf*GUS L2 and *Tf*GUS mL1) and an L1 from *Treponema lecithinolyticum* (*Tl*GUS L1). We determined the x-ray crystal structures of *Tl*GUS L1, *Tf*GUS mL1, and *Tf*GUS L2 to 1.6, 2.2, and 2.3 Å resolution, respectively (table S1). The central fold consisting of two β-sandwich–like domains (red and yellow, fig. S2) and the active site-containing TIM barrel (gray, fig. S2) are conserved, while the quaternary structures, confirmed with size exclusion chromatography–multi-angle light scattering (SEC-MALS) (fig. S3), were found to vary (Fig. 2). *Tl*GUS L1 and *Tf*GUS mL1 are distinct homotetramers, with *Tf*GUS mL1 further containing a C-terminal domain of unknown function (DUF4982) (Fig. 2 and fig. S2B). *Tf*GUS L2 is a homodimer with both DUF4982 and malectin-like domains at its C terminus (fig. S2D).

The L1 and L2 regions are distinct among the proteins examined here (Fig. 2). *Tl*GUS L1 exhibits the smallest and most sterically constricted active sites (fig. S4A). A portion of its 380-403 L1 region is ordered, while residues Gly^386^-Lys^395^ of this loop are disordered, and residues Ser^166^-Thr^177^ from neighboring monomers swap into each active site (fig. S5B). The active site of *Tf*GUS L2 is larger but still constrained by aromatic side chains, while *Tf*GUS mL1 offers an open catalytic gorge (fig. S4, B and C) akin to the accessibility observed for human GUS (fig. S4D). These results highlight the structural variability present in GUS enzymes from the human oral microbiome. They suggest that the smaller active sites created by some of these microbial enzymes may lead to enhanced activity with the small reporter substrates used as a biomarker for periodontitis, and the more accessible active sites may process periodontal-related polysaccharides.

### Oral microbial GUS enzymes can degrade periodontal glucuronide-containing polysaccharides

During periodontitis, bacteria break down polysaccharides within the periodontal tissues and disseminate to sites distant from the oral cavity (*29*). Major components of the periodontium include the connective tissue extracellular matrix, alveolar bone, and periodontal ligament, all of which contain chondroitin sulfate (CS), a glycosaminoglycan with alternating sugars of GlcA and N-acetylgalactosamine (GalNAc). It has been shown that GUS enzyme orthologs from the human intestinal microbiome catalyze the removal of terminal nonreducing β-d-glucuronides of heparan (*22*), making polysaccharides from the periodontium a potential substrate for oral microbial GUS proteins.

**Fig. 3.**
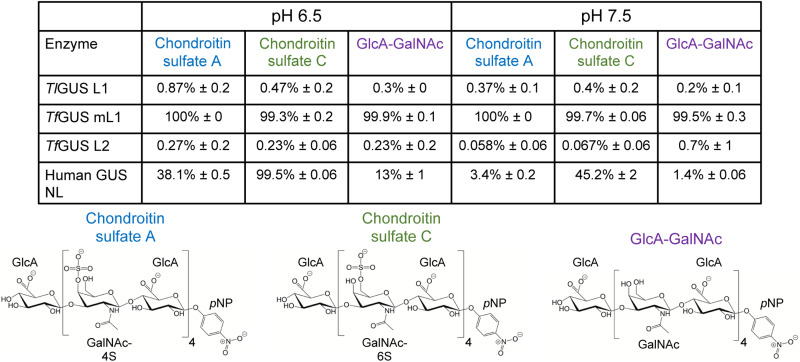
Processing of various CS compounds by GUS at pH 6.5 and 7.5. Errors represent the SD of *N* = 3 technical replicates.

Polysaccharide processing is performed outside microbial cells; thus, we first examined the GUS proteins identified from the oral microbiome for the presence of signal sequences that would facilitate extracellular trafficking. We found that all mL1, L2, and NTL GUS enzymes from the oral microbiome contained a predicted N-terminal signaling sequence (data S1) (*30*), indicating that these GUS enzymes have the potential to engage with polysaccharide substrates in the extracellular milieu. Thus, we tested the ability of the oral microbial GUS proteins in-hand to process glucuronide-containing constituents of the periodontium and compared the results to the activity of human GUS, which is known to degrade CS (*11*). We evaluated the cleavage of the terminal β-d-glucuronide from different synthetic CS-like polysaccharides under two physiological pH values relevant to the periodontal sulcus—6.5 and 7.5. In periodontitis, the pH of the sulcus increases from 6.5 to 7.5. 9-mers of CS-A, CS-C, and a nonsulfated polymer of GlcA and GalNAc were examined (Fig. 3). CS-A and CS-C are major components of the periodontium, and their breakdown products are elevated in clinical sites from active periodontitis individuals (*31*).

We found that human GUS showed low activity for GlcA-GalNAc, intermediate activity for CS-A, and high activity for CS-C, as well as consistently higher activity at pH 6.5 than at pH 7.5 for all substrates (Fig. 3). In addition, we found that while *Tl*GUS L1 and *Tf*GUS L2 exhibited less than 1% processing activity for any compound regardless of pH, *Tf*GUS mL1 showed essentially 100% processing of all substrates independent of pH. These data demonstrate that human GUS differentially processes CS-like polysaccharides in a manner dependent on pH and compound structure. By contrast, the mL1 GUS from *T. forsythia* exhibited robust activities on all substrates at both pH 6.5 and 7.5. These data indicate that mL1 GUS proteins from the oral microbiome can participate in extracellular matrix degradation, thus contributing to periodontal disease etiology.

We next focused on the mL1 GUS proteins from the oral microbiome. We found that all seven arise from genera associated with periodontitis: *Prevotella*, *Bacteroides*, or *Tannerella*. To evaluate the potential for other mL1 GUS enzymes to process polysaccharides with similar efficiency to *Tf*GUS mL1, we predicted the structures of the unique mL1 GUS using AlphaFold2 (*32*). First, the AlphaFold2 prediction for *Tf*GUS mL1 was aligned to the experimental structure described here. The root mean square deviation (RMSD) for all atoms was 0.55 Å, indicating good agreement between this experimental structure and the in silico model (table S2). Second, structural alignments of all mL1 GUS AlphaFold2 models revealed a similar tertiary structure (all RMSD range 0.20 to 0.69 Å) and conservation of the active site architecture (fig. S6). Third, a multiple sequence alignment shows that the mL1 region, defined as 378 to 386 in *Tf*GUS mL1, is almost completely conserved across all mL1 enzymes (fig. S7). Fourth, the Tyr^380^ that uniquely projects into the *Tf*GUS mL1 active site is completely conserved in all mL1 orthologs in the oral microbiome. On the basis of these structural and sequence alignments, it is expected that the mL1 enzymes in the human oral microbiome, which are encoded by periodontitis-associated taxa, will exhibit the same activities against CS-like polysaccharides demonstrated for *Tf*GUS mL1. Thus, the degradation of glycosaminoglycans in the periodontium, which facilitates microbial dissemination to distant sites, appears to be a consequence of both human and oral microbial GUS enzymes and that microbial enzymes are more efficient at this process in conditions associated with worsening periodontal disease.

### Biomarker processing is driven by oral microbial GUS enzymes

GUS activity is elevated in both the GCF and in saliva collected from individuals with periodontitis, and this has been used as a biomarker for the disease (*12*, *13*). This activity has to this point been assumed to arise solely from the human GUS released by degranulating neutrophils. For this reason, GUS activity is traditionally measured using a glucuronide-containing reporter substrate at pH conditions between 4.5 and 5.0, the optimal range for human GUS (*33*). However, the pH of GCF ranges from 6.5 to 8.5, with higher values present during active and severe periodontitis (*34*). To determine whether oral microbial GUS enzymes could process a glucuronide-containing reporter substrate, we performed in vitro activity assays using 4-methylumbelliferyl-β-d-glucuronide (4-MUG) under varying pH conditions and compared the human GUS enzyme to the three oral microbial GUS enzymes purified here.

Differential processing of 4-MUG was observed (Table 1 and figs. S8 to S11). At pH 5.0, *Tl*GUS L1 and *Tf*GUS mL1 were more catalytically efficient than human GUS, a trend that becomes even more pronounced at physiological pH values ranging from 6.5 to 7.5. The catalytic efficiency of the human GUS enzyme was 122-fold higher at its optimal pH of 5.0 than at 7.5. Furthermore, the catalytic efficiencies of *Tl*GUS L1 and *Tf*GUS mL1 were 8000- and 140-fold higher than the human form at pH 7.5. Thus, oral microbial GUS enzymes are orders-of-magnitude more efficient at glucuronide-containing reporter substrate processing than the human GUS, particularly at the higher pH values found in periodontal disease. These findings suggest that clinical measures of GUS activity in periodontitis are heavily influenced by oral microbial enzymes rather than those generated by the human host, even at the pH value of 5 often used in testing.

**Table 1.** Kinetic constants for 4-MUG hydrolysis by various GUS enzymes at pH 5.0, 6.5, 7.0, and 7.5. Errors represent the SEM of *N* = 3 technical replicates. NA, no activity.

	*T. lecithinolyticum* GUS L1			*T. forsythia* GUS mL1			*T. forsythia* GUS L2			Human GUS (NL)		
pH	*k*_cat_ (s^−1^)	*K*_m_ (μM)	*k*_cat_/*K*_m_ (s^−1^ M^−1^)	*k*_cat_ (s^−1^)	*K*_m_ (μM)	*k*_cat_/*K*_m_ (s^−1^ M^−1^)	*k*_cat_ (s^−1^)	*K*_m_ (μM)	*k*_cat_/*K*_m_ (s^−1^ M^−1^)	*k*_cat_ (s^−1^)	*K*_m_ (μM)	*k*_cat_/*K*_m_ (s^−1^ M^−1^)
5.0	19 ± 1	6.8 ± 1	2.8 × 10^6^	8.5 ± 0.3	8.0 ± 2	1.1 × 10^6^	NA	NA	NA	2.2 ± 0.05	84 ± 7	2.6 × 10^4^
6.5	172 ± 9	61 ± 9	2.8 × 10^6^	14 ± 0.6	21 ± 4	6.7 × 10^4^	0.094 ± 0.007	300 ± 56	313	1.0 ± 0.02	2746 ± 587	364
7.0	161 ± 8	73 ± 8	2.2 × 10^6^	14 ± 0.3	26 ± 2	5.4 × 10^4^	0.30 ± 0.1	497 ± 220	603	0.90 ± 0.15	3062 ± 704	294
7.5	238 ± 36	140 ± 35	1.7 × 10^6^	12 ± 0.6	42 ± 6	2.9 × 10^4^	0.21 ± 0.05	221 ± 79	950	0.72 ± 0.11	3380 ± 668	213

### UNC4917 is an inhibitor of the *T. lecithinolyticum* L1 GUS

**Fig. 4.**
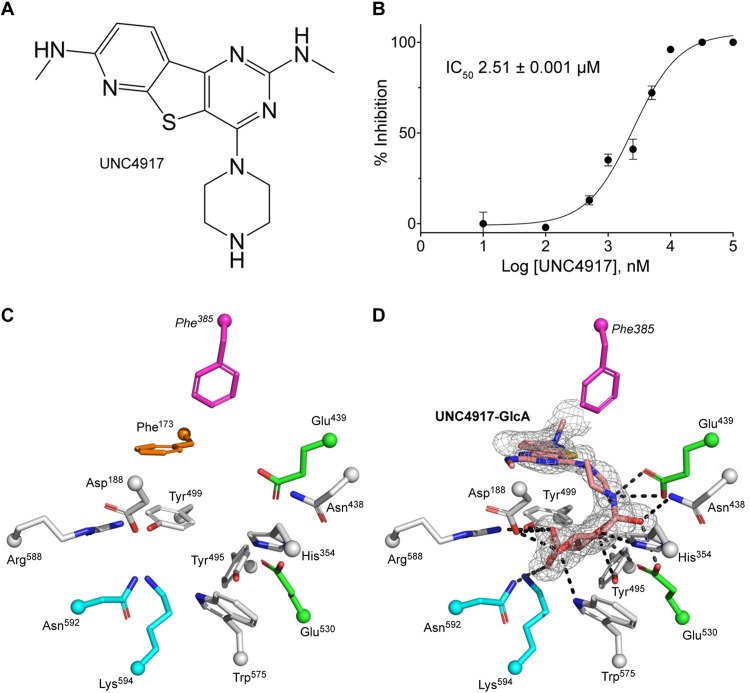
UNC4917 is a potent inhibitor of *Tl*GUS, an L1 GUS. (**A**) Chemical structure of UNC4917. (**B**) IC_50_ determination of UNC4917 for *Tl*GUS using the 4-MUG assay. Errors represent the SD of *N* = 3 technical replicates. (**C**) Active site conformation of the apo structure of *Tl*GUS. The catalytic glutamate amino acids are colored green, and the NxK motif amino acids required for GlcA orientation in the active site are colored cyan. Phe^385^ (pink) is projecting from the L1 region, and Phe^173^ (orange) is extending from a neighboring monomer. (**D**) Active site conformation of *Tl*GUS with the UNC4917-GlcA conjugate bound (salmon). Dashed lines represent hydrogen bonding distances between UNC4917-GlcA and residues within the active site. The electron density associated with UNC4917-GlcA was generated from simulated-annealing (*F*_obs_ − *F*_calc_) omit maps and is contoured at 3σ.

To further understand the role of microbial GUS enzymes in periodontal disease, we sought to examine their behavior when exposed to an inhibitor selective for microbial GUS proteins. L1 microbial GUS enzymes have been shown to be potently inhibited by small molecules that uniquely intercept the catalytic cycle to form a distinct inhibitor-glucuronide conjugate (*18*, *35*). These inhibitors are selective for microbial GUS proteins and do not affect human GUS (*18*). We examined *Tl*GUS L1 with the L1 GUS inhibitor UNC4917 using the 4-MUG hydrolysis assay (Fig. 4, A and B). Enzyme kinetic progress curves for *Tl*GUS L1 with UNC4917 exhibited a nonlinear pattern (fig. S12), a feature consistent with previously reported slow-binding characteristics for UNC4917 with human gut microbial L1 GUS enzymes (*18*). Using the initial linear rate, we determined the half-maximal inhibitory concentration (IC_50_) for UNC4917 to be 2.5 μM for *Tl*GUS L1 (Fig. 4B). We also found that UNC4917 failed to inhibit *Tf*GUS mL1, *Tf*GUS L2, or human GUS (fig. S13).

To confirm that UNC4917 intercepts the catalytic cycle to form a distinct UNC4917-glucuronide conjugate, we crystallized *Tl*GUS L1 in the presence of *p*-nitrophenyl-glucuronide (PNPG) and UNC4917 and determined the resultant crystal structure to 1.75-Å resolution (Fig. 4, C and D). PNPG is a GUS substrate and initiates the catalytic cycle of the enzyme, which UNC4917 then intercepts. The structure revealed the formation of a covalent β linkage between the anomeric carbon of GlcA and the secondary piperazine nitrogen of UNC4917 (fig. S14). The UNC4917-GlcA conjugate is located within the active site (fig. S5B) and forms an edge-to-face π-π stacking with Phe^385^ (Fig. 4D). These results establish that UNC4917 intercepts the catalytic cycle of an L1 GUS from the human oral microbiome. We further determined the 1.95-Å-resolution crystal structure of *Tl*GUS L1 in the presence of PNPG and ciprofloxacin, an antibiotic that is effective in the treatment of periodontitis and is known to be highly concentrated in the GCF (*36*, *37*). Ciprofloxacin, like UNC4917, contains a secondary amine-containing piperazine and exhibits an IC_50_ of 18.3 μM for *Tl*GUS L1 (fig. S15A). The *Tl*GUS L1 crystal structure with ciprofloxacin shows that ciprofloxacin also intercepts the catalytic cycle to form a covalent β linkage between its secondary amine and the anomeric carbon of GlcA (fig. S15B). Thus, oral microbiome–encoded L1 GUS enzymes are subject to unique and selective inhibition by piperazine-containing drugs, such as ciprofloxacin and the synthetic inhibitor UNC4917.

### GUS activity from oral clinical samples is reduced by UNC4917

As noted above, GUS activity is increased in GCF samples obtained from individuals with periodontitis, and this activity serves as a biomarker for disease severity (*12*). Since periodontitis involves both host immune cell infiltrates and gingival microbial dysbiosis, we hypothesized that clinical measured GUS activity is a composite of the human and microbial GUS enzymes. To test this hypothesis, we obtained GCF samples from 23 individuals diagnosed with untreated periodontitis stages I to III (table S3). These samples were collected using sterile PerioPaper strips from the two deepest periodontal sites located in the two most severely involved dental quadrants, and 4-MUG cleavage activity was measured for each sample in the presence and absence of the microbial-specific GUS inhibitor UNC4917. This approach yielded a data structure of sample dyads (level 1) nested within periodontal sites (level 2) nested again within individuals (level 3), resulting in a total of eight samples per patient. A three-level mixed-effects model with bootstrapping was used to examine GUS activity [in micromolar 4-methylumbelliferone (MU) per hour] as a function of GUS inhibition by UNC4917, along with disease severity measured by periodontal probing depth (PPD) and periodontitis stage.

**Fig. 5.**
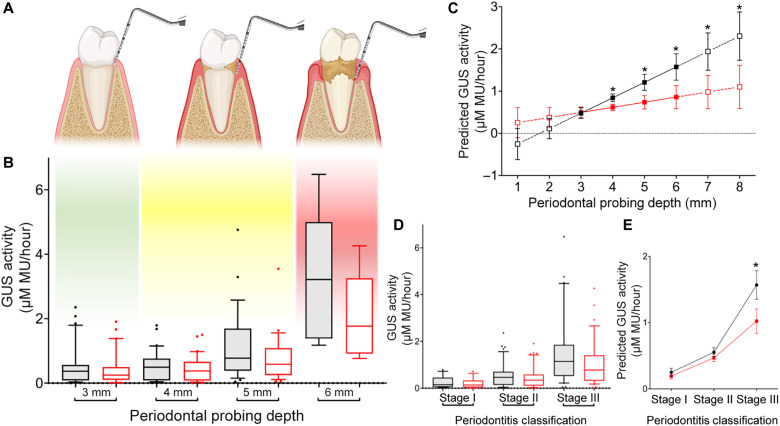
GUS activity from oral clinical samples increases with periodontitis severity, and this activity is inhibited by UNC4917. (**A**) Depiction of periodontal health status with associating PPD. (**B**) GUS activity, as determined by the 4-MUG assay, from GCF samples obtained from untreated sites with various PPDs in the presence (red) and absence (black) of UNC4917 (*n* = 92). (**C**) Predicted GUS activity with PPD (in millimeters) in the presence (red) and absence (black) of UNC4917 with 95% confidence interval (nonoverlapping confidence intervals indicate statistical significance, *P* < 0.05). (**D**) Periodontitis stage as it relates to GUS activity in the presence and absence of UNC4917. (**E**) Predicted GUS activity with periodontitis stage in the presence and absence of UNC4917 with 95% confidence interval (nonoverlapping confidence intervals indicate statistical significance, *P* < 0.05). Boxplots: Lower, middle, and upper bounds correspond to the 25th, 50th, and 75th percentiles, respectively. Upper and lower whiskers extend to the 90th and 10th percentiles, respectively.

The local environment of the periodontal sulcus favors a dysbiotic microbiota and onset of inflammation as PPD worsens. Therefore, we determined whether GUS activity from GCF samples, as measured by 4-MUG cleavage, is directly proportional to PPD. 4-MUG cleavage activity increased as PPD increased from 3 to 6 mm (Fig. 5B). Furthermore, addition of UNC4917, which does not inhibit human GUS but does inhibit 4-MUG cleavage by oral microbial GUS enzymes, demonstrated a reduction in GUS activity with increasing PPD (Fig. 5C and table S4). On the basis of the GUS activity prediction model, UNC4917 shows minimal inhibition of GUS activity at 3-mm PPD sites but significantly reduces activity at sites with PPDs ranging from 4 to 8 mm (Fig. 5C). Last, when 4-MUG cleavage activity was considered as a function of periodontitis staging, a direct relationship was observed between increased periodontitis stage and enhanced GUS inhibition by UNC4917 (Fig. 5, D and E). Thus, the increase in clinical GUS activity that accompanies periodontitis severity is driven by GUS proteins from the oral microbiome. This observation advances our understanding of disease etiology and may lead to novel personalized diagnostic and treatment avenues.

## DISCUSSION

Severe periodontal disease affects ~10% of the global population and disproportionally affects minorities in the United States (*38*, *39*). Left untreated, the persistent inflammatory burden associated with periodontitis influences the management of other systemic diseases and reduces overall quality of life (*5*, *40*). The pathogenesis of periodontal disease progresses from a microbial challenge to a secondary host response. Although more than 90 components from GCF have been examined as clinical indicators of periodontitis severity, to date, no single biomarker has been shown to capture both the microbial and host contributions to disease etiology (*34*, *41*, *42*). The well-established GCF GUS activity assay, first described in 1970, has been assumed to arise solely from human GUS released by degranulating neutrophils (*43*). The past decade has established that the human GI microbiome encodes genes for >3000 GUS proteins and that this diverse family of microbial enzymes directly influences gut and systemic health (*14*–*17*, *22*, *24*, *35*, *44*–*48*). Thus, we hypothesized that GCF GUS activity arose from both the human GUS and from enzymes generated by the oral microbiome.

The GUS atlas mapped here from the human oral microbiome contains 165 total and 53 unique GUS enzymes, with many being produced by periodontal disease–associated genera, such as *Tannerella*, *Treponema*, *Prevotella*, and *Fusobacterium*, and is notably distinct from the GUS proteins found in the GI tract (*22*, *49*). NL enzymes represented the majority (54%) of the GUS genes found in the human gut, followed by mL1 (15%), L2 (14%), L1 (6%), mL2 (4%), and mL1,2 (2%) (*14*). By contrast, L1 GUS enzymes are the majority (58%) in the oral microbiome, followed by 15% NL, 13% mL1, and less than 10% for the remaining classes. Only the pathogenic *T. forsythia* species is found to contain an L2 GUS gene in the oral microbiome. Furthermore, while 81% of the L1 enzymes in the gut are encoded by Firmicutes and only 6% by Proteobacteria, these distributions are notably altered in the oral cavity, with 42% of L1 GUS from Proteobacteria and 41% from Firmicutes. The distinctions between oral and gut GUS enzymes likely reflect differences in substrate availability. In contrast to the GI tract, dietary polysaccharides are transiently present in the oral cavity and have limited access to the subgingival environment (*50*). Thus, the oral microbiome appears to require far fewer NL GUS proteins that have been shown to be capable of acting on larger polysaccharides (*22*).

The anaerobic and pathogenic genera *Prevotella* and *Tannerella* encode mL1 GUS enzymes with predicted signaling sequences for periplasmic localization (*30*). Breakdown products of the periodontal extracellular matrix, which include glucuronide-containing glycosaminoglycans such as hyaluronate and CS, are found at periodontitis sites (*51*). The efficient processing of CS compounds by *Tf*GUS mL1 suggests that it contributes to degradation of the periodontal extracellular matrix. Furthermore, the succession of bacteria during periodontal disease development is accompanied by the degradation of host proteins, release of ammonia from amino acid metabolism, and an increase in pH (*34*). GCF from the periodontal sulcus of periodontitis sites have pH values approaching 8, suggesting a microenvironment that is not favorable for the human GUS, a lysosomal protein with a pH optimum of 5 (*52*). The presence of oral microbial GUS enzymes efficiently active at basic pH values indicates that pathogenic bacterium such as *T. forsythia*, a red complex bacterium, disrupts the periodontal extracellular matrix integrity during periodontitis. Despite *T. forsythia* having an asaccharolytic physiology, our data demonstrate that it encodes multiple GUS proteins active against small and large glucuronide-containing substrates (*53*). Other phylogenetically related microbes, such as *Bacteroides thetaiotaomicron*, have polysaccharide utilization loci (PUL) capable of scavenging host-derived saccharides when dietary polysaccharides are absent (*54*). *T. forsythia* contains a PUL-like operon, which may enhance its in vivo fitness during periodontitis development (*55*). Further studies will be required to fully elucidate the role of these microbial GUS enzymes and potential mutualism in the establishment of a dysbiotic microbiota. However, the results presented here indicate that oral microbial GUS proteins can promote human periodontal disease, particularly by degrading the extracellular matrix of periodontal tissues.

During periodontal inflammation, neutrophils migrate to the oral mucosal barrier, transmigrate into the periodontal sulcus, and release a variety of signaling molecules and enzymes, including human GUS, in response to the microbial challenge at this barrier (*9*, *56*). Measuring GUS activity from oral samples with reporter glucuronide substrates is well established (*10*, *13*, *57*–*63*) and has high diagnostic value for periodontitis (*12*, *64*). However, human GUS exhibits poor catalytic efficiency against the reporter substrate 4-MUG compared to the oral microbial GUS enzymes examined here. The large human GUS active site limits binding of small glucuronide-containing molecules and reduces catalytic efficiency. Therefore, measuring GUS activity from oral samples at a neutral pH using small glucuronide compounds is likely dominated by oral microbial GUS enzymes rather than human GUS. Furthermore, our data show that oral microbial enzymes are superior at reporter substrate processing even at lower pH values, indicating the key role that these nonhuman enzymes play in this diagnostic measure of periodontal disease.

We show that increased GUS activity from GCF is directly associated with clinical parameters, including increased PPD and severity of periodontitis. We further demonstrate a significant reduction in GUS activity in GCF samples by UNC4917, a microbial GUS-specific inhibitor, indicating that microbial GUS enzymes contribute directly to the enhanced activity in disease. In addition, as periodontitis severity increased, so did the percent inhibition of clinical GUS activity by UNC4917. Thus, the GUS activity in the periodontal sulcus is a direct measure of the microbiota in periodontitis. This finding is particularly important because nucleotide-based sequencing methodologies are incapable of detecting the functions of microbial gene products, which, in the case of bacterial GUS activity, is a marker of periodontal disease progression. Furthermore, periodontitis severity is associated with an increase in microbial community diversity of the subgingival microbiome (*26*). Many periodontitis-associated genera, such as *Prevotella*, *Tannerella*, and *Treponema*, have GUS enzymes within the L1 and mL1 classes, which are highly efficient at processing small glucuronide reporter substrates. Thus, GUS activity may indiscriminately capture the diverse microbial signatures associated with periodontitis.

In summary, we present an atlas of GUS proteins in the oral microbiome and, through structurally guided inhibition studies, redefine the origin of GUS activity in clinical periodontal disease samples. The actualization that the GUS activity biomarker for periodontitis captures both the pathogenic microbes and the host’s immune response represents an important advance in our understanding of the etiology of this prevalent disease. As a result, closely monitoring GUS activity may predict periodontitis initiation and future disease progression (*65*), a topic that is especially critical as the global burden of periodontitis has remained unchanged for decades (*39*). Furthermore, the appreciation that oral microbial GUS enzymes can advance the degradation of connective tissue polysaccharides suggests potential treatment avenues for blocking disease progression. Future efforts will be directed toward determining whether UNC4917 can be developed as a potential therapeutic for the treatment of periodontitis. Together, the findings outlined here may enable more personalized and effective therapies for periodontitis and enhance our appreciation of the roles microbial enzymes play in degrading protective barrier tissues in inflammatory disease.

## MATERIALS AND METHODS

### Study design

The objective of this study was to define a biomarker for periodontitis that captures both the host and microbial activity in a single clinical measurement. We hypothesized that GUS activity from oral samples satisfies our objective and emphasized this concept through the following experimental methodologies: (i) outline the diversity of microbial GUSs within the oral microbiome, (ii) compare the processing efficacy of microbial and human GUS for common polysaccharides found within the periodontium, (iii) compare the processing efficacy of microbial and human GUS for glucuronide-containing reporter substrates, and (iv) determine the contribution of microbial GUS enzymes to GUS activity from oral clinical samples obtained from individuals with untreated periodontitis. The eHOMD was used to identify the diversity of GUSs from oral microbes. Key representative microbial GUSs were subsequently selected from pathogenic bacteria to characterize both kinetically and structurally. Crystal structures highlighted the unique features of various microbial GUSs. Glucuronide-containing polysaccharides commonly found in the periodontium were obtained, and polysaccharide processing for both microbial and human GUS was determined. The crystal structures provided insight into how the microbial GUSs were superior at processing glucuronide-containing polysaccharides. A glucuronide-containing reporter substrate (4-MUG) was then used to compare the activity of microbial GUSs to the human GUS and the kinetic characterization of a unique inhibitor (UNC4917) selective against microbial GUSs. Oral clinical samples were obtained, and UNC4917 was used to demonstrate that GUS activity increases with disease severity and that this increase in activity is influenced by a microbial GUS contribution. A power analysis was not calculated, and all data points were included for each clinical sample.

### Human oral microbial GUS atlas identification

A combined protein sequence FASTA file from the expanded eHOMD v3 was downloaded from http://homd.org/ftp/genomes/PROKKA/current/faa/, and GUSs were identified from the examined protein FASTA files using a previously established bioinformatics approach (*22*). In brief, reference GUS sequences from *E. coli* [(*Ec*GUS); UniProt: P05804, Protein Data Bank (PDB): 3LPF], *S. agalactiae* (*Sa*GUS; UniProt: Q8E0N2, PDB: 4JKL), *C. perfringens* (*Cp*GUS; UniProt: Q8VNV4, PDB: 4JKM), and *B. fragilis* (*Bf*GUS; PDB: 3CMG) were each aligned pairwise to protein sequences from eHOMD v3 using Protein-Protein BLAST (BLASTP v2.5.0+) (*66*). Candidate sequences with ≥25% identity to any representative GUS were then assessed for the presence of seven conserved residues (*22*). Specifically, we filtered for protein sequences containing conserved active site residues located at the following positions for each reference sequence: *Ec*GUS: N412, E413, Y468, E504, N566, K568, and G569; *Bf*GUS: N411, E412, Y468, E505, N567, K569, and G570; *Sa*GUS: N407, E408, Y464, E501, N563, K565, and G566; and *Cp*GUS: N428, E429, Y479, E510, N581, K583, and G584. Sequences that both met the identity threshold and contained all seven conserved residues were accepted as GUS enzymes. Accepted sequences were then filtered for redundancies at a sequence identity threshold of 90% using CD-HIT (v4.8.1), and the output was used to form a representative set of GUS sequences for downstream analysis (*67*). Accepted sequences were aligned to representative sequences from each loop category in a multiple sequence alignment, and GUS category was assigned according to parameters reported previously (*19*, *20*, *22*). Representative sequences were clustered using the EMBL-EBI search, which was combined with the GUS category to create cladograms using ggtree (v3.2.1) and ggplot2 (v3.3.5) in R (v4.1.2) (*68*–*71*).

### Recombinant protein cloning, expression, and purification

The GH2 genes identified from *T. forsythia* (SEQF2738_02236; SEQF2738_01199) and *T. lecithinolyticum* (SEQF2462_01804) were codon-optimized for *E. coli* expression and subcloned into a pET (His)_6_ LIC (2Bc-T) expression vector by Bio Basic Inc. (Amherst, NY) (data S1). The protein sequences were evaluated for the presence of an N-terminal targeting sequence using SignalP 6.0, and this tag was omitted during the cloning process where indicated (*30*). The correct gene sequence of each construct was confirmed by complete gene sequencing by Bio Basic Inc.

Protein production and purification was performed as previously described (*14*, *19*, *22*). Briefly, all proteins were expressed in *E. coli* BL21 DE3 Gold cells. Cells were cultured in 1.5 liters of LB media containing ampicillin (50 μg/ml) at 37°C. At an optical density (600 nm) of 0.6, the temperature was adjusted to 18°C, and isopropyl β-d-1-thiogalactopyranoside was added to a final concentration of 0.1 mM. Cell growth continued overnight at 18°C. The following day, cells were pelleted via centrifugation, drop-frozen in liquid nitrogen, and stored at −80°C until protein purification.

All proteins were purified using Ni^2+^ affinity and size exclusion gel filtration chromatography. Harvested cells were resuspended in buffer A containing 20 mM potassium phosphate (pH 7.4), 500 mM NaCl, and 50 mM imidazole. Deoxyribonuclease, lysozyme, and a Roche cOmplete EDTA-free protease inhibitor tablet were added to the suspension immediately before sonication. Cells were disrupted by sonication, and the cell lysate was cleared by centrifugation at 4°C and a 0.22-μm filter before loading onto a 5-ml Ni-nitrilotriacetic acid HP column (GE Healthcare). The protein was subsequently washed with 10 ml of buffer A and eluted with buffer B containing 20 mM potassium phosphate (pH 7.4), 500 mM NaCl, and 250 mM imidazole. Purified protein was pooled and subjected to size exclusion chromatography (HiLoad 16/600 Superdex 200 gel filtration column) using buffer C [20 mM Hepes (pH 7.4) and 50 mM NaCl]. The N-terminal (His)_6_ tag was retained for all forms of protein purified. Purified protein was concentrated to 10 to 15 mg/ml, flash-frozen in liquid nitrogen, and stored at −80°C. The only exception was *Tf*GUS mL1, which precipitated out of solution during concentration. *Tf*GUS mL1 was therefore repurified, as described above, with the most prominent elution peak from the size exclusion column drop-frozen in liquid nitrogen and stored at −80°C. The recombinant human GUS enzyme (Leu^23^-Thr^651^) was purified from mouse myeloma cell line to ensure posttranslational modification and purchased from R&D Systems (Minneapolis, MN).

### SEC-MALS analysis of oral GUS proteins

*Tf*GUS mL1, *Tf*GUS L2, and *Tl*GUS L1 were analyzed on a Superdex 200 size exclusion column connected to an Agilent fast protein liquid chromatography system, Wyatt DAWN HELEO II multi-light scattering instrument, and a Trex refractometer. Each protein was diluted to 2 mg/ml in 20 mM Hepes (pH 7.4) and 300 mM NaCl buffer, and a volume of 50 μl was injected onto the column. A flow rate of 0.5 ml/min was used for all proteins. Light scattering and refractive index data were collected and analyzed using Wyatt ASTRA (version 8.1) software. A specific refractive index increment (dn/dc) value of 0.185 ml/g was used for calculations.

### Steady-state kinetic assays for GUS using 4-MUG

All reactions were carried out in a Costar black 96-well clear flat bottom plate at 25°C. The reaction conditions consisted of a buffer system (50 mM) and 50 mM NaCl (final concentrations). Various buffer systems were used for each pH of interest, and these buffers consisted of sodium acetate (pH 4.5 and 5.0), MES (pH 6.5), MOPS (pH 7.0), and Hepes (pH 7.5). The total reaction volume was 50 μl, each reaction was initiated by the addition of 4-MUG, and the final concentration of enzyme in the reaction mixture was specific to each GUS: 35 nM human GUS, 5 nM *Tl*GUS L1, 11 to 24 nM *Tf*GUS mL1, and 800 nM *Tf*GUS L2. Reactions were monitored continuously using a CLARIOstar microplate reader with an excitation wavelength of 350 nm and an emission wavelength of 450 nm. The linear portion of the progress curve was determined by a custom linear regression analysis program in MATLAB, and initial velocities were analyzed using GraphPad Prism 9. Data were fit to either the Michaelis-Menten or substrate inhibition equation to determine *k*_cat_ and *K*_m_.

### UNC4917 synthesis and IC_50_ determination for UNC4917

As previously reported, UNC4917 was synthesized at the University of North Carolina (UNC) at Chapel Hill Center for Integrative Chemical Biology and Drug Discovery (*18*). In vitro inhibition of GUS activity by UNC4917 was screened for each enzyme using the 4-MUG hydrolysis assay. Reactions were performed in a Costar black 96-well plate clear flat bottom plate at 25°C. Reaction conditions consisted of 50 mM MOPS (pH 7.0), 50 mM NaCl, 1 mM 4-MUG, varying concentrations of UNC4917, and GUS (final concentrations). Final enzyme concentrations used were as follows: 35 nM human GUS, 5 nM *Tl*GUS L1, 10 nM *Tf*GUS mL1, and 800 nM *Tf*GUS L2. The total reaction volume was 50 μl, and the enzyme was preincubated with UNC4917 for 5 min before initiating the reaction with 4-MUG. Reactions were monitored continuously using a CLARIOstar microplate reader with an excitation wavelength of 350 nm and an emission wavelength of 450 nm. The linear portion of the progress curve was determined by a custom linear regression analysis program in MATLAB. Initial velocities were converted to % inhibition values via the following equation

$$\%\mathrm{Inhibition}=\left[1-\frac{{\left(\mathrm{RFU}\cdot s^{-1}\right)}_{\exp}-{\left(\mathrm{RFU}\cdot s^{-1}\right)}_{\mathrm{bg}}}{{\left(\mathrm{RFU}\cdot s^{-1}\right)}_{\mathrm{no}\;\mathrm{inhib}}-{\left(\mathrm{RFU}\cdot s^{-1}\right)}_{\mathrm{bg}}}\right]$$

where (RFU · s^−1^)_exp_ is the rate at a particular inhibitor concentration, (RFU · s^−1^)_no inhib_ is the uninhibited reaction, and (RFU · s^−1^)_bg_ is the background rate. Calculated percent inhibitions were then plotted against the log of inhibitor concentration and fit using a four-parameter logistic function with GraphPad Prism 9 to determine the concentration at which IC_50_ is observed.

### Protein crystallization, data collection, and structure determination

All proteins were crystallized via the hanging drop vapor diffusion technique at room temperature. For *Tf*GUS L2, a protein solution consisting of 11.5 mg/ml was mixed at a 1:1 ratio with a precipitant solution composed of 6% (w/v) polyethylene glycol 8000 (PEG8000), 8% (v/v) ethylene glycol, 2% (v/v) glycerol, and 0.1 M tris-HCl (pH 8.8). Clustered rod-shaped crystals grew to full size within 2.5 weeks. The *Tf*GUS mL1 protein crystallized under similar conditions where the protein solution (6.3 mg/ml) was mixed at a 1:1 ratio with the precipitant solution consisting of 6% (w/v) PEG8000, 12% (v/v) ethylene glycol, 5% (v/v) glycerol, and 0.1 M tris-HCl (pH 8.5). Apo crystals of *Tl*GUS L1 formed after mixing the protein solution (14.6 mg/ml) at a 1:1 ratio with the precipitant solution consisting of 2 M ammonium sulfate, 0.1 M MOPS (pH 7.0), and 4% 2-methyl-2,4-pentanediol. For the structure of *Tl*GUS L1 with UNC4917-GlcA bound, the protein was incubated with 1 mM UNC4917 and 2.25 mM PNPG for 30 min before setting up the crystallization conditions. The protein-ligand solution was subsequently mixed at a 1:1 ratio with a precipitant solution composed of 2.4 M ammonium sulfate and 0.1 MOPS (pH 7.0). For the structure of TlGUS L1 with ciprofloxacin-GlcA bound, the protein was incubated with 1.5 mM ciprofloxacin and 2.25 M PNPG for 30 min and then mixed with a solution containing 2.4 M ammonium sulfate and 0.1 M MOPS (pH 7.0) at a 1:1 ratio.

The *Tf*GUS crystals were cryoprotected by transferring the protein crystals to a well solution supplemented with ethylene glycol to a final concentration of 20% (v/v) and then flash-cooled in liquid nitrogen. The apo and ligand-bound crystals of *Tl*GUS L1 were both flash-cooled in liquid nitrogen directly from the crystallization drop.

X-ray diffraction data were collected on the 23-ID-D beamline at the General Medical Sciences and Cancer Institute’s Structural Biology Facility (Advanced Photon Source, Argon National Laboratory). Diffraction images were integrated with DIALS (*72*) or XDS (*73*) and scaled with AIMLESS (*74*). Structure solution for all proteins was achieved by molecular replacement using PHASER with the search model PDB codes 5UJ6, 3CMG, and 6BJQ for *Tf*GUS L2, *Tf*GUS mL1, and *Tl*GUS L1, respectively (*75*). Initial models were built using the Autobuild pipeline within the Phenix suite followed by several rounds of manual model building with COOT (*76*). Successive refinements were performed with Phenix.refine (*77*). Structural analysis and figures were generated using PyMOL (version 2.5.2). The data collection and refinement statistics are summarized in table S1, and all structures reported are available in the PDB as accessions, 8DHE, 8DHL, 8DHV, 8DHW, and 8E72.

### CS processing assay

All CS-PNP (CS; 9-mer) compounds (>98% purity) contained a GlcA residue at the terminal (nonreducing) position and were obtained from Glycan Therapeutics (Raleigh, NC). The CS compounds were incubated with each GUS enzyme for 30 min at 37°C. Reaction conditions consisted of 0.5 μM GUS enzyme and 10 μg of the CS compound. All reactions were performed in triplicate and terminated by heating at 95°C for 10 min. Each GUS enzyme exhibited no activity following heat denaturing (fig. S16). Reaction aliquots were analyzed by ProPac high-performance LC with a strong anion exchange column (Propac PA1, 10 μm, 9 × 250 mm, Thermo Fisher Scientific) on the Shimadzu Prominence UFLC20A instrument (Shimadzu Corporation). A total of 80 μl of each quenched reaction was injected on the column with a flow rate of 1 ml/min, and the ultraviolet monitor was set at 310 nm. Buffer A consisted of 20 mM NaAcO (pH 5.0), and buffer B was 20 mM NaAcO (pH 5.0) with 2 M NaCl. For the CS-A compound, a linear gradient from 0 to 40% buffer B was programmed from 0 to 3 min followed by a gradient of 40 to 60% buffer B from 3.1 to 30 min. The linear gradients for the CS-C compound were from 0 to 50% buffer B in 0 to 3 min followed by 50 to 70% buffer B in 3.1 to 30 min. The linear gradients for the CS base compound were from 0 to 10% buffer B in 0 to 3 min followed by 10 to 30% buffer B in 3.1 to 40 min. The CS cleavage activity for each GUS enzyme was reported in percent CS cleavage by integrating the start material and product peaks using the standard references of start materials and the digested products.

### Collection of oral clinical samples and GUS activity determination

Samples were obtained from the biospecimen repository at the UNC Adams School of Dentistry. All GCF samples were collected under study protocol Institutional Review Board (IRB) no. 10-1159 and deidentified for use in this study. Briefly, a total of 23 study participants were recruited to the General and Oral Health Center—UNC Adams School of Dentistry and represent a convenience cohort. Inclusion criteria included adults (18 years or older) with a minimum of six opposing teeth in a functional dentition. Individuals were excluded if they required antibiotic prophylaxis before dental care, presented with a fixed orthodontic appliance, reported being pregnant or nursing, or had any medical condition where dental treatment was contraindicated. Once study participants provided informed consent, all individuals underwent full mouth comprehensive periodontal charting, including PPD and clinical attachment level. At visit 2, two GCF samples, which were predetermined from the periodontal charting, were collected from interproximal sites with the deepest periodontal pockets in each quadrant. To obtain the GCF samples, the teeth were isolated and dried. Supragingival plaque was removed with sterile curettes, and a sterile PerioPaper strip (Oraflow Inc.) was placed at the base of periodontal sulcus for 30 s. Despite our best efforts, the GCF samples may contain bacteria. PerioPaper strips were removed, immediately flash-frozen in liquid nitrogen, and stored at −80°C until analyzed. All participants were diagnosed with periodontitis (stages I to III) from the comprehensive periodontal charting obtained at visit 1 (*75*).

GCF samples were resuspended in 100 μl of buffer containing 50 mM MOPS (pH 7.0) and 50 mM NaCl. Each sample was divided into two aliquots to determine GUS activity in the presence and absence of UNC4917. The first reaction contained 1 mM 4-MUG (final concentration), while the paired reaction contained 1 mM 4-MUG and 100 μM UNC4917 (final concentration). The total reaction volume was 50 μl, and all reactions were incubated at 37°C for 6 hours. Each reaction was quenched by heating to 95°C for 10 min. Reactions were then allowed to cool to room temperature, and 10 μl of 0.5 M *N*-cyclohexyl-3-aminopropanesulfonic acid (CAPS) (pH 10.5) was immediately added to each reaction to enhance the fluorescence intensity. A total of 50 μl of each reaction sample was transferred into a Costar black 96-well clear flat bottom plate and evaluated using a CLARIOstar microplate reader with an excitation wavelength of 360 nm and an emission wavelength of 450 nm.

### Statistical analysis

Statistical analyses of the GCF sample data were conducted using Stata 15. The data structure was of sample dyads (level 1) nested within periodontal sites (level 2) nested within individuals (level 3). To account for the nested structure of the data, we tested three-level mixed-effects models using bootstrapping of the SEs with 100 iterations. The intercept-only model yielded intraclass correlations of 0.56, 0.14, and 0.31 for levels 1, 2, and 3, respectively. All data points from the collected clinical samples were included in the statistical analysis.
