# Supplementary material for: Microbial β-glucuronidases drive human periodontal disease etiology
Source: Sci Adv. 2023 May 5;9(18):eadg3390. doi: 10.1126/sciadv.adg3390 (PMC10162664; doi:10.1126/sciadv.adg3390)
Supplement: Supplementary file 1 — Figs. S1 to S16 Tables S1 to S4 Legend for data S1 [file sciadv.adg3390_sm.pdf]

Science Advances
AAAS

# Supplementary Materials for

## Microbial β-glucuronidases drive human periodontal disease etiology

Adam D. Lietzan *et al.*

Corresponding author: Matthew R. Redinbo, redinbo@unc.edu

*Sci. Adv.* **9**, eadg3390 (2023)
DOI: 10.1126/sciadv.adg3390

**The PDF file includes:**

Figs. S1 to S16
Tables S1 to S4
Legend for data S1

**Other Supplementary Material for this manuscript includes the following:**

Data S1

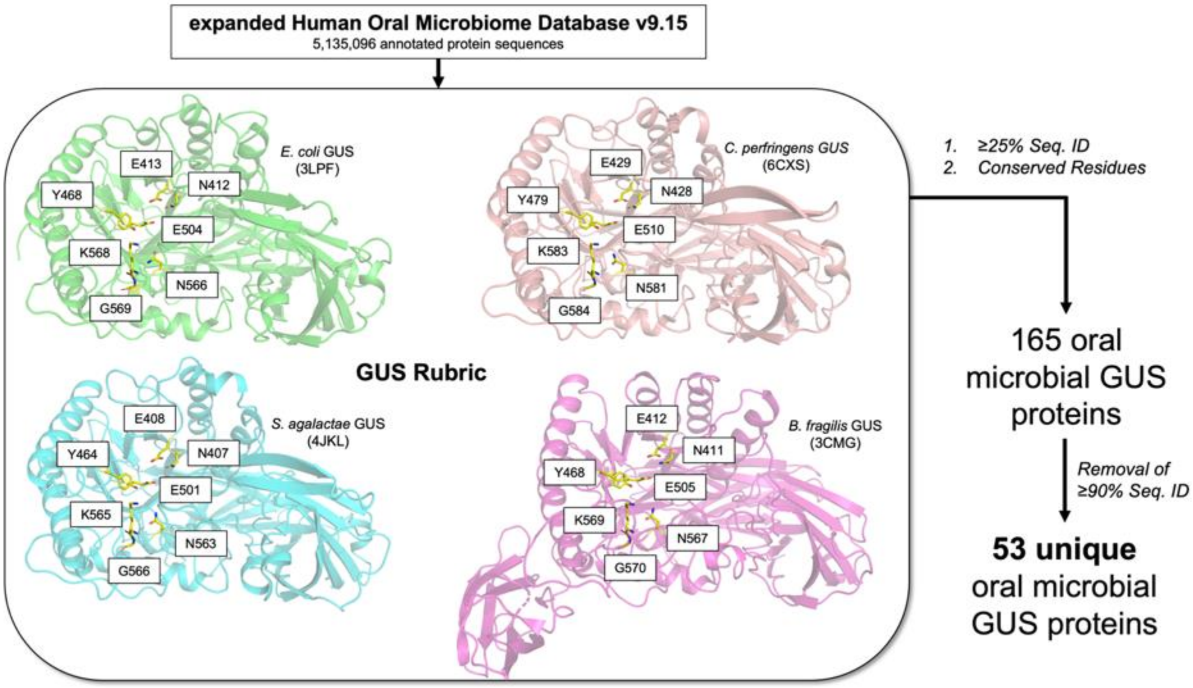


**Fig. S1.**

**Schematic outlining the rubric used to define the oral microbial β-glucuronidase atlas from the human oral microbiome.**

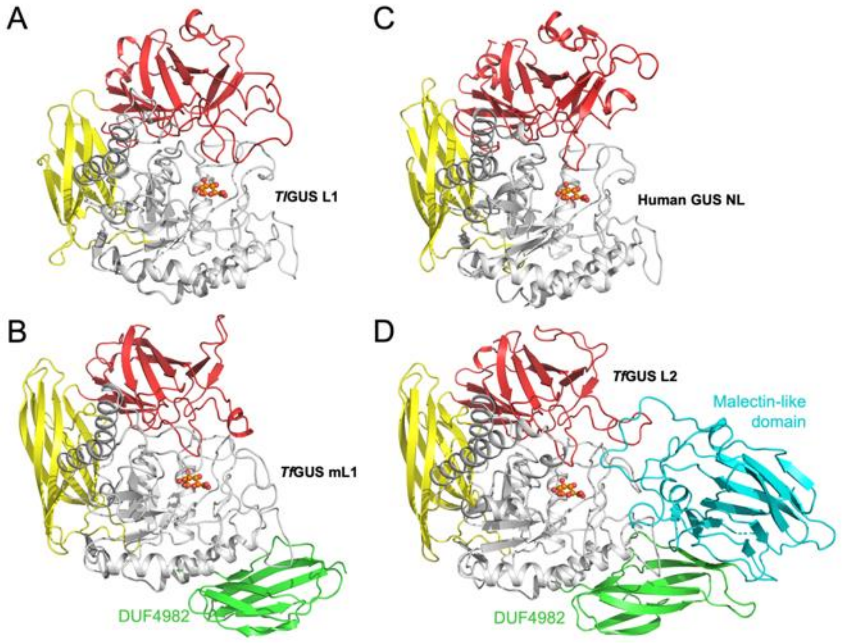


**Fig. S2.**

**Tertiary structure of various GUS enzymes. (A)** Monomer of *Tl*GUS L1. **(B)** Monomer of *Tf*GUS mL1. **(C)** Monomer of human GUS. **(D)** Monomer of *Tf*GUS L2. The core fold is defined as two beta-sandwich-like domains (red, yellow) and the active site-containing TIM barrel fold (grey). GlcA (orange) has been modeled into the active site for orientation purposes. DUF4982 is colored green and the malectin-like domain is colored cyan.

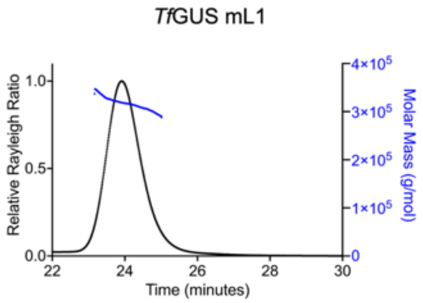


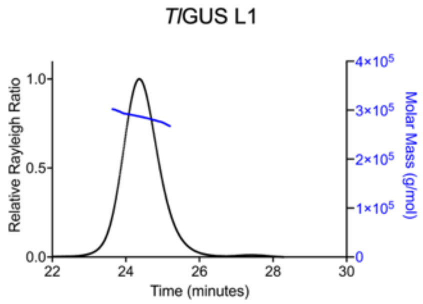


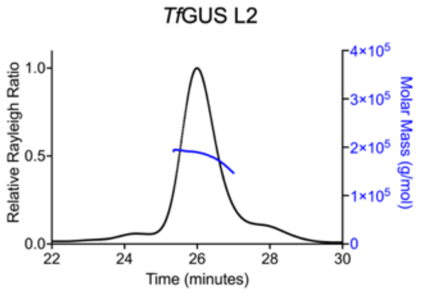


| GUS enzyme | Molar mass (g/mol) | Oligomeric state |
|---|---|---|
| *Tf*GUS mL1 | $3.177 \times 10^5$ | Tetramer |
| *Tf*GUS L2 | $1.839 \times 10^5$ | Dimer |
| *Tl*GUS L1 | $2.880 \times 10^5$ | Tetramer |

**Fig. S3.**

**Multi-angle light scattering coupled with size exclusion chromatography (SEC-MALS) for the selected oral microbial GUS enzymes.**

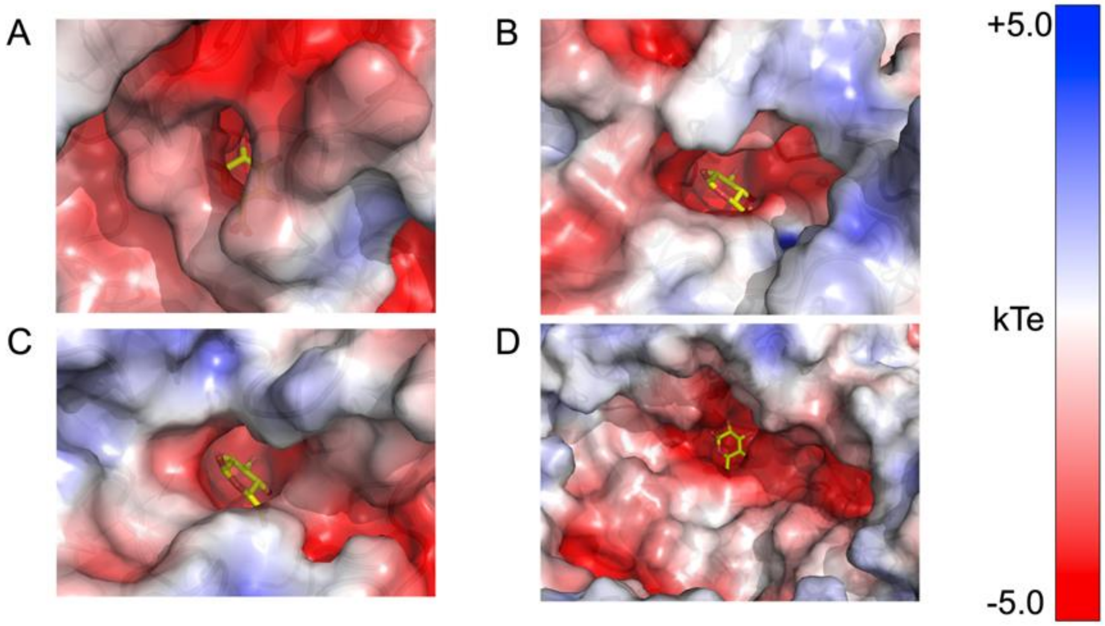


**Fig. S4.**
**Electrostatic potential surface rendering of the active sites of various GUS enzymes.** **(A)** *Tl*GUS L1. **(B)** *Tf*GUS mL1. **(C)** *Tf*GUS L2. **(D)** Human GUS. GlcA (yellow) was modeled into the active site of each enzyme based upon previous GUS structures with GlcA bound.

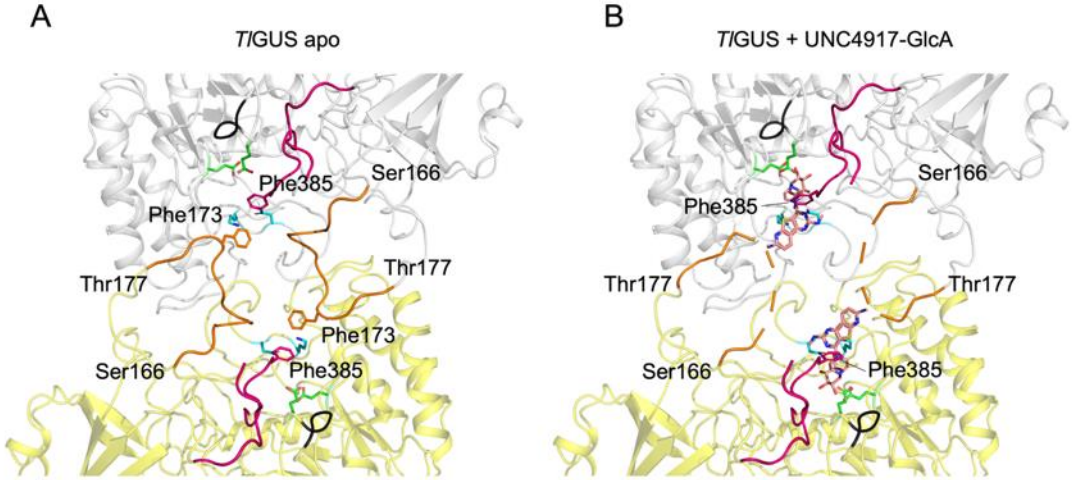


**Fig. S5.**
**Homodimer interface and active sites of *Tl*GUS L1 with (B) and without (A) UNC4917-GlcA conjugate bound.**

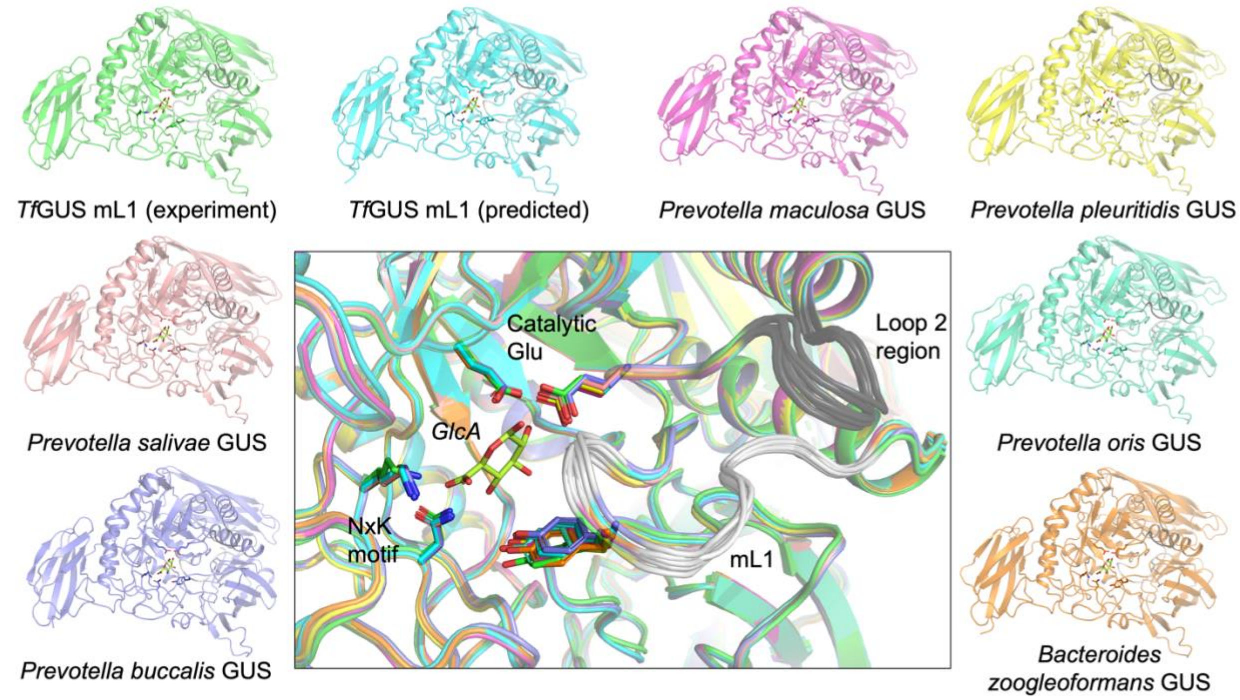


**Fig. S6.**
**The AlphaFold2 predicted mL1 GUS proteins from the oral microbiome are near identical to the experimentally determined *Tf*GUS mL1 structure.** The mL1 region is colored white except for the Tyr that is projecting towards the active site. The loop 2 region is colored dark grey. GlcA was modeled into the active site for orientation purposes.

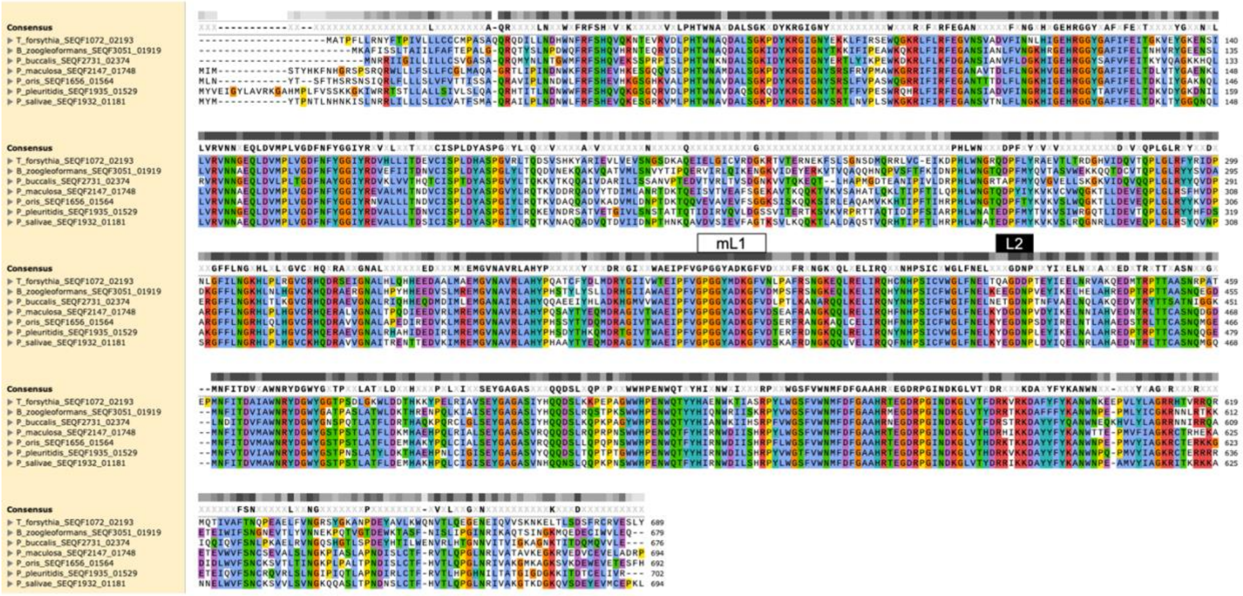

**Fig. S7.**
**Multiple sequence alignment of all unique mL1 GUS from the oral microbiome.** The mL1 (white box) and L2 regions (black box) are highlighted within the sequence alignment.

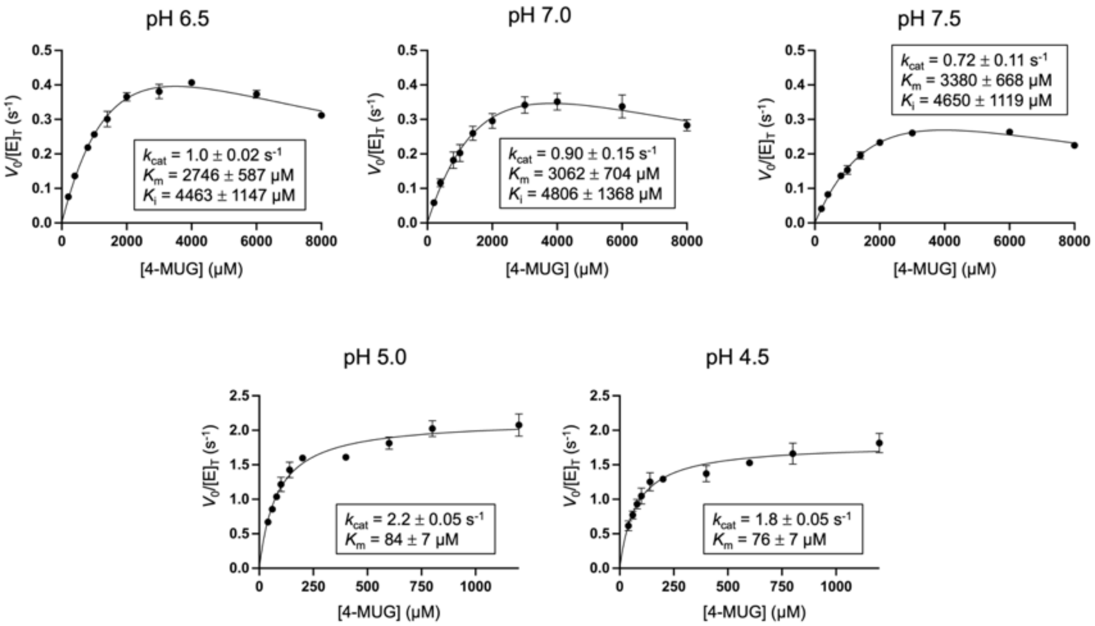


**Fig. S8.**

**4-MUG hydrolysis by the human GUS enzyme at pH 4.5, 5.0, 6.5, 7.0, and 7.5**. Data from pH 4.5 and 5.0 were fit to the Michaelis-Menten equation using non-linear regression. At pH 6.5 - 7.5, data were fit to the substrate inhibition equation using non-linear regression. Reaction conditions: rhGUS wt [35 nM], 50 mM buffer system consisting of sodium acetate (pH 4.5 or 5.0), MES (pH 6.5), MOPS (pH 7.0), or HEPES (pH 7.5), 50 mM NaCl, 25ºC. Data points were measured in triplicate and error bars are standard deviations. The pH optima for rhGUS was previously reported to be 4.5.

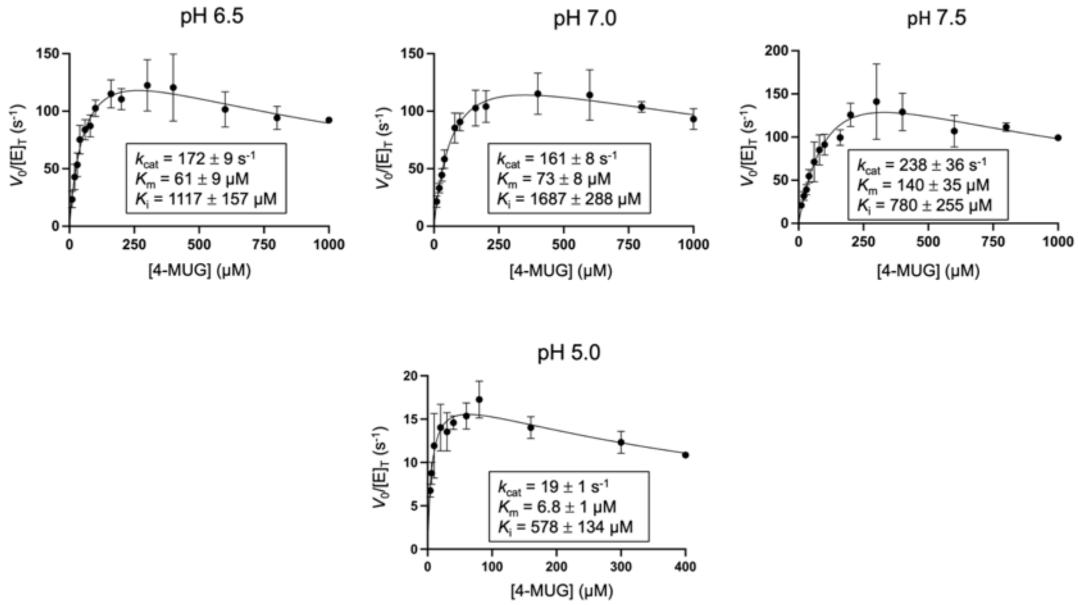


**Fig. S9.**
**4-MUG hydrolysis by the *T. lecithinolyticum* GUS (L1) enzyme at pH 5.0, 6.5, 7.0, and 7.5.** All data were fit to the substrate inhibition equation using non-linear regression. Reaction conditions: *Tl*GUS L1 [5 nM], 50 mM buffer system consisting of sodium acetate (pH 5.0), MES (pH 6.5), MOPS (pH 7.0), or HEPES (pH 7.5), 50 mM NaCl, 25ºC. Data points were measured in triplicate and error bars are standard deviations.

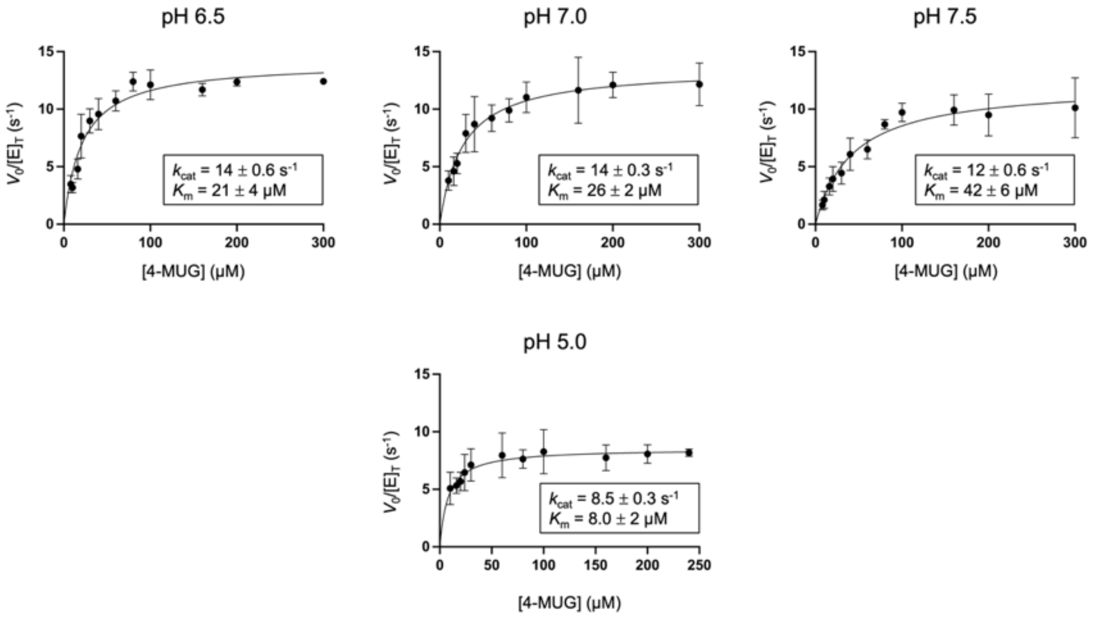


**Fig. S10.**
**4-MUG hydrolysis by the *T. forsythia* GUS (mL1) enzyme at pH 5.0, 6.5, 7.0, and 7.5.** All data were fit to the Michaelis-Menten equation using non-linear regression. Reaction conditions: *Tf*GUS (mL1) [11-24 nM], 50 mM buffer system consisting of sodium acetate (pH 5.0), MES (pH 6.5), MOPS (pH 7.0), or HEPES (pH 7.5), 50 mM NaCl, 25ºC. Data points were measured in triplicate and error bars are standard deviations.

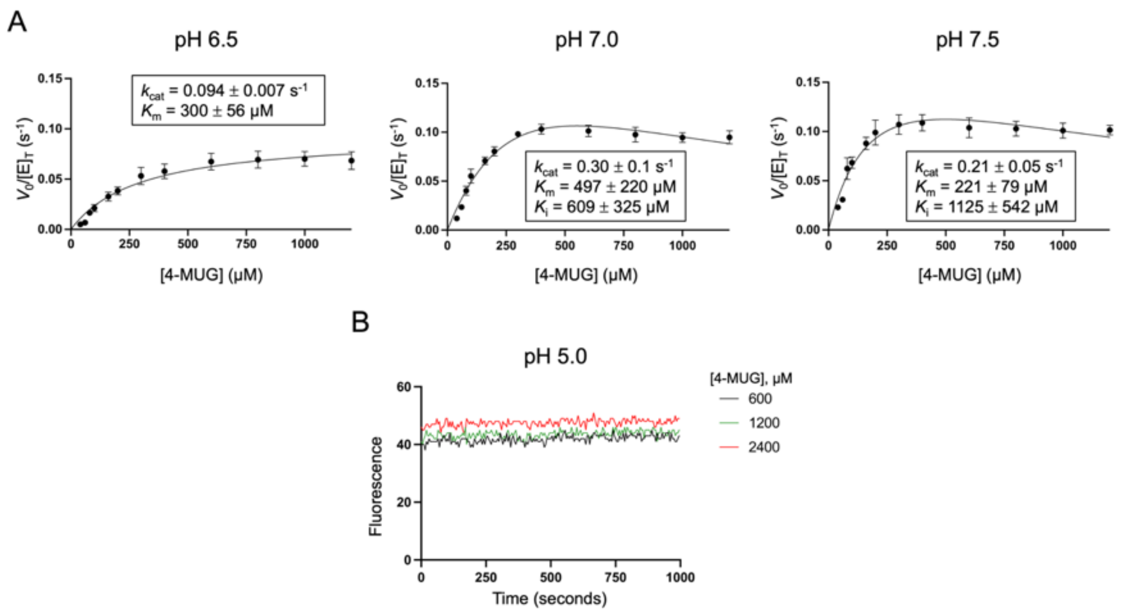


**Fig. S11.**
**4-MUG hydrolysis by the *T. forsythia* GUS (L2) enzyme at pH 5.0, 6.5, 7.0, and 7.5.** **(A)** Data collected at pH 6.5 were best fit to the Michaelis-Menten equation using non-linear regression. Data collected at pH 7.0 and 7.5 exhibited substrate inhibition and therefore were best fit to the substrate inhibition equation using non-linear regression. Reaction conditions: *Tf*GUS-L2 [800 nM], 50 mM buffer system consisting of MES (pH 6.5), MOPS (pH 7.0), or HEPES (pH 7.5), 50 mM NaCl, 25ºC. Data points were measured in triplicate and error bars are standard deviations. **(B)** No 4-MUG hydrolysis activity was observed at varying concentrations of 4-MUG at pH 5.0 (50 mM sodium acetate). Reaction conditions were identical to those reported above.

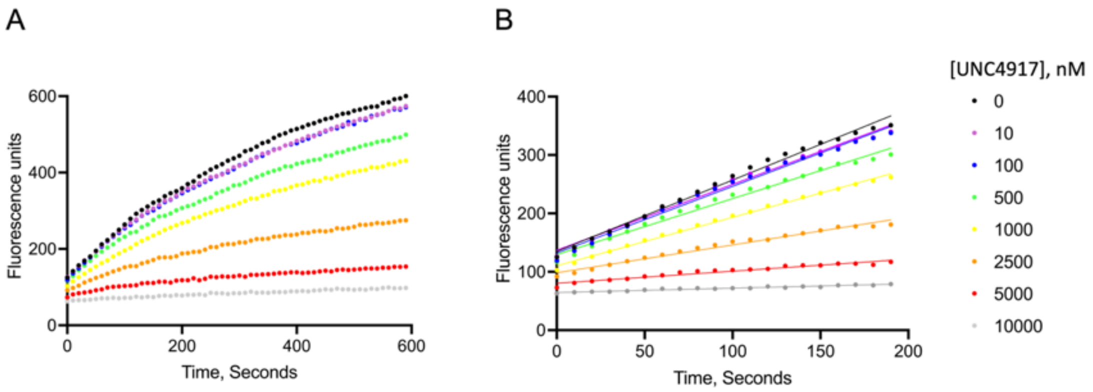


**Fig. S12.**
***T. lecithinolyticum* GUS (L1) exhibits non-linear enzyme kinetic progress curves in the presences of UNC4917. (A)** Enzyme kinetic progress curves for 4-MUG hydrolysis in the presence of increasing concentrations of UNC4917. **(B)** Linear regression fits ($r^2 > 0.95$) during the linear portion of the enzyme kinetic progress curve. Linear regression fits were generated using MATLAB.

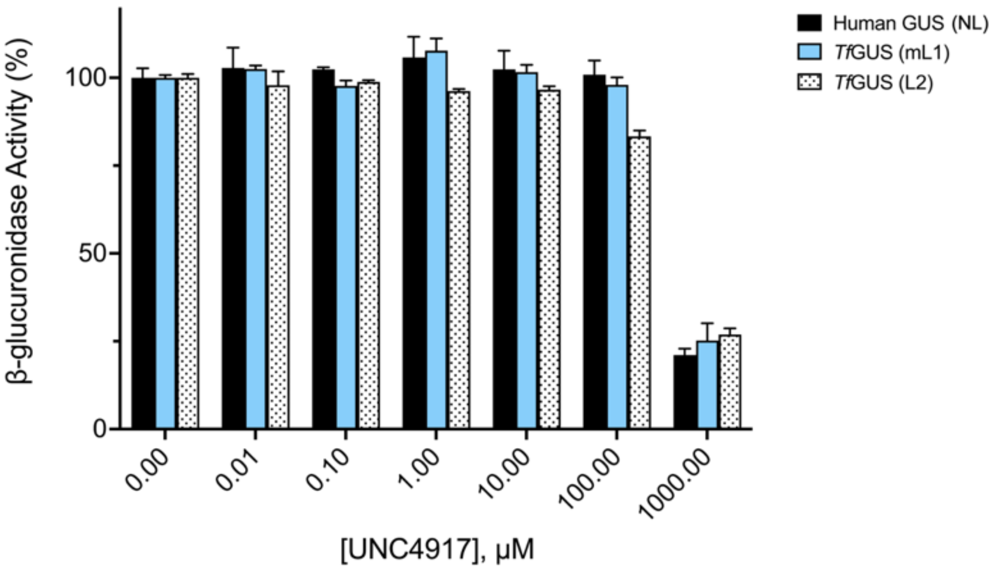


**Fig. S13**
**Percent catalytic activities for the hydrolysis of 4-MUG by human GUS and *T. forsythia* (mL1 and L2) in the presence of varying concentrations of the inhibitor UNC4917.** Reaction conditions were as follows: 50 mM MOPS (pH 7.0), 50 mM NaCl, 1 mM 4-MUG, 25ºC. Protein concentrations were as follows: human GUS [35 nM], *Tf*GUS mL1 [10 nM], and *Tf*GUS L2 [800 nM]. Error bars represent standard deviation using N = 3.

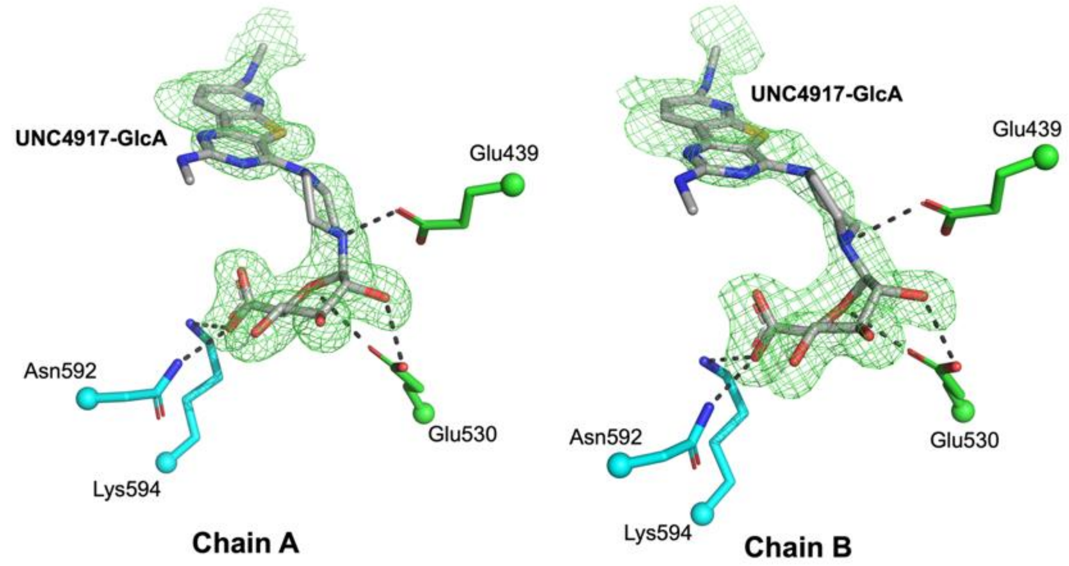


**Fig. S14.**
**Electron density of the UNC4917-GlcA conjugate in the active site of *Tl*GUS L1 for each monomer in the asymmetric unit.** The catalytic glutamates are colored green and the amino acids for the NxK motif are colored cyan. The electron density (green mesh, contoured at 3σ) for the ligands was generated from simulated-annealing ($F_o$-$F_c$) omit maps.

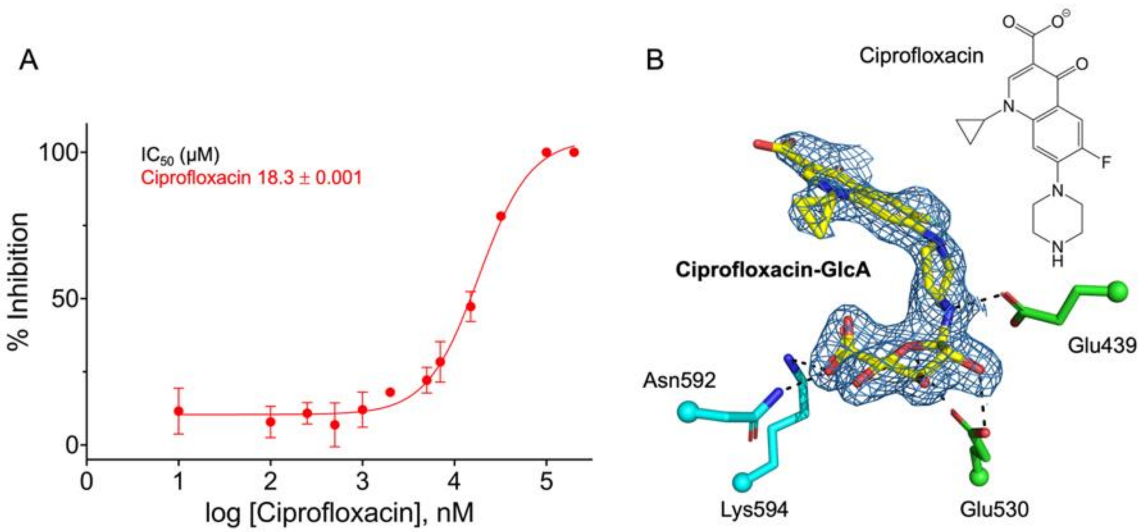


**Fig. S15.**

**The oral microbial GUS from *T. lecithinolyticum* is inhibited by ciprofloxacin. (A)** $IC_{50}$ determination of ciprofloxacin for *Tl*GUS L1 using the 4-MUG assay. Errors represent the SD of $N = 3$ replicates. **(B)** Electron density of the ciprofloxacin-GlcA conjugate in the active site of *Tl*GUS L1. The catalytic glutamates are colored green and the amino acids for the NxK motif are colored cyan. The $2F_o$-$F_c$ electron density (blue mesh) is contoured at $1\sigma$.

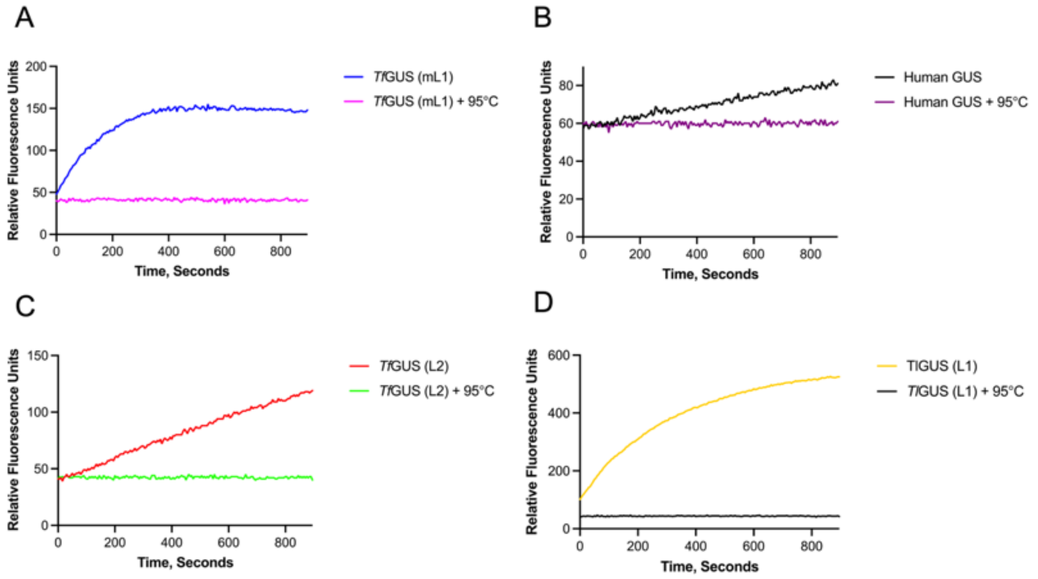


**Fig. S16.**
**4-MUG hydrolysis by GUS enzymes with and without heat inactivation. (A)** *T. forsythia* GUS (mL1) [25 nM], 100 µM 4-MUG. **(B)** Human GUS [35 nM], 3 mM 4-MUG. **(C)** *T. forsythia* GUS (L2) [800 nM], 600 µM 4-MUG. **(D)** *T. lecithinolyticum* GUS (L1) [5 nM], 250 µM 4-MUG. Reaction conditions: 50 mM MES (pH 6.5), 50 mM NaCl, 25ºC. Enzymes were incubated at 95ºC for 10 minutes prior to initiating the reaction.

**Table S1. Crystallography data collection and refinement statistics.**

| | *T. forsythia* GUS mL1 | *T. forsythia* GUS L2 | *T. lecithinolyticum* GUS | *T. lecithinolyticum* GUS + UNC4917-GlcA | *T. lecithinolyticum* GUS + ciprofloxacin-GlcA |
|---|---|---|---|---|---|
| PDB code | 8DHE | 8DHL | 8DHV | 8DHW | 8E72 |
| Wavelength (Å) | 1.03318 | 0.96802 | 1.03318 | 1.03319 | 1.03320 |
| Resolution range (Å) | 49.70 - 2.20 (2.28 - 2.20) | 78.77 - 2.30 (2.38 - 2.30) | 48.92 - 1.60 (1.66 - 1.60) | 48.94 - 1.75 (1.81 - 1.75) | 66.76 - 1.95 (2.02 - 1.95) |
| Space group | P 1 21 1 | P 21 21 21 | P 41 21 2 | P 41 21 2 | P 41 21 2 |
| a, b, c (Å) | 100.44, 139.65, 109.82 | 77.98, 119.21, 209.88 | 94.30, 94.30, 287.95 | 94.49, 94.49, 287.61 | 94.41, 94.41, 288.01 |
| α, β, γ (°) | 90, 94.501, 90 | 90, 90, 90 | 90, 90, 90 | 90, 90, 90 | 90, 90, 90 |
| Total reflections | 288,317 (28,244) | 575,048 (56,836) | 1,896,661 (98,797) | 1,317,506 (54,345) | 821,651 (84,679) |
| Unique reflections | 147,774 (14,626) | 87,470 (8,591) | 170,770 (16,829) | 131,384 (12,533) | 94,825 (9,438) |
| Multiplicity | 2.0 (1.9) | 6.6 (6.6) | 11.1 (5.9) | 10.0 (4.3) | 8.7 (9.0) |
| Completeness (%) | 96.69 (96.01) | 99.77 (99.59) | 99.48 (99.45) | 99.48 (96.42) | 98.79 (99.95) |
| Mean I/σ(I) | 6.9 (1.1) | 7.0 (3.4) | 13.2 (0.9) | 14.5 (1.1) | 9.9 (1.0) |
| Wilson B factor ($Å^2$) | 33.4 | 22.2 | 23.7 | 27.1 | 35.9 |
| $R_{merge}$ (%) | 9.5 (67.6) | 19.3 (53.6) | 9.2 (164.0) | 8.7 (117.7) | 14.8 (251.5) |
| $R_{meas}$ (%) | 12.8 (91.9) | 20.9 (58.2) | 9.6 (180.1) | 9.2 (133.6) | 15.7 (266.7) |
| $R_{pim}$ (%) | 8.6 (61.9) | 8.1 (22.5) | 2.8 (73.2) | 2.8 (61.7) | 5.3 (87.3) |
| $CC_{1/2}$ | 0.993 (0.554) | 0.979 (0.844) | 0.999 (0.603) | 0.999 (0.531) | 0.998 (0.463) |
| CC* | 0.998 (0.844) | 0.995 (0.957) | 1 (0.867) | 1 (0.833) | 1 (0.796) |
| **Refinement Statistics** | | | | | |
| Resolution range (Å) | 51.71 - 2.20 (2.26 - 2.20) | 78.77 - 2.30 (2.36 - 2.30) | 49.15 - 1.60 (1.64 - 1.60) | 49.13 - 1.75 (1.79 - 1.75) | 66.76 - 1.95 (2.00 - 1.95) |

| | | | | | |
|---|---|---|---|---|---|
| Reflections used in refinement | 147,735 (14,613) | 87,447 (8,590) | 170,474 (16,770) | 131,159 (12,509) | 94,682 (9,434) |
| Reflections used for $R_{free}$ | 2,007 (205) | 2,000 (197) | 1,997 (195) | 1,995 (190) | 1,994 (199) |
| $R_{cryst}$ | 0.205 (0.326) | 0.167 (0.209) | 0.167 (0.322) | 0.173 (0.275) | 0.184 (0.293) |
| $R_{free}$ | 0.244 (0.365) | 0.207 (0.297) | 0.196 (0.332) | 0.212 (0.296) | 0.212 (0.333) |
| $CC_{work}$ | 0.957 (0.687) | 0.955 (0.906) | 0.969 (0.820) | 0.968 (0.807) | 0.969 (0.763) |
| $CC_{free}$ | 0.939 (0.609) | 0.938 (0.850) | 0.962 (0.821) | 0.951 (0.764) | 0.963 (0.704) |
| Number of non-hydrogen atoms | 22,005 | 14,047 | 10,723 | 10,541 | 10,245 |
| macromolecules | 21,214 | 13,227 | 9,652 | 9,498 | 9,520 |
| ligands | 52 | 42 | 89 | 125 | 92 |
| solvent | 739 | 778 | 982 | 918 | 633 |
| Protein residues | 2,637 | 1,680 | 1,195 | 1,179 | 1,194 |
| RMS bonds (Å) | 0.009 | 0.008 | 0.007 | 0.007 | 0.008 |
| RMS angles (°) | 1.03 | 0.90 | 0.91 | 1.01 | 1.08 |
| Ramachandran favored (%) | 95.66 | 97.12 | 96.21 | 96.40 | 96.11 |
| Ramachandran allowed (%) | 4.15 | 2.82 | 3.79 | 3.60 | 3.89 |
| Ramachandran outliers (%) | 0.19 | 0.06 | 0.00 | 0.00 | 0.00 |
| Rotamer outliers (%) | 0.59 | 1.47 | 1.38 | 1.01 | 0.91 |
| Clashscore | 6.12 | 4.13 | 2.36 | 2.88 | 3.38 |
| Average B-factor (Å$^2$) | 37.28 | 22.21 | 29.58 | 30.86 | 38.49 |
| Macromolecules (Å$^2$) | 37.34 | 22.06 | 28.79 | 30.06 | 38.20 |

| | | | | | |
|---|---|---|---|---|---|
| Ligands (Å$^2$) | 40.04 | 29.40 | 44.71 | 42.18 | 48.82 |
| Solvent (Å$^2$) | 35.16 | 24.32 | 35.98 | 37.63 | 41.34 |

Statistics for the highest-resolution shell are shown in parentheses.

**Table S2.**

**Structural alignment and sequence identity of mL1 GUS family members.** RMSD values are reported in units of Å for the alignment of all atoms (white cells). Shaded cells correspond to percent identity between primary sequences.

| | *Tf*GUS mL1 exp | *Tf*GUS mL1 AlphaFold2 | *Prevotella maculosa* GUS | *Prevotella pleuritis* GUS | *Prevotella oris* GUS | *Prevotella salivae* GUS | *Prevotella buccalis* GUS | *Bacteroides zoogleoformans* GUS |
|---|---|---|---|---|---|---|---|---|
| *Tf*GUS mL1 exp | - | 0.545 | 0.686 | 0.676 | 0.668 | 0.593 | 0.579 | 0.600 |
| *Tf*GUS mL1 AlphaFold2 | 100% | - | 0.457 | 0.407 | 0.492 | 0.398 | 0.415 | 0.348 |
| *Prevotella maculosa* GUS | 57.44% | - | - | 0.286 | 0.218 | 0.245 | 0.427 | 0.344 |
| *Prevotella pleuritis* GUS | 59.55% | - | 71.83% | - | 0.294 | 0.284 | 0.43 | 0.317 |
| *Prevotella oris* GUS | 57.96% | - | 79.33% | 69.46% | - | 0.199 | 0.388 | 0.338 |
| *Prevotella salivae* GUS | 57.19% | - | 75.58% | 70.02% | 82.05% | - | 0.334 | 0.273 |
| *Prevotella buccalis* GUS | 59.22% | - | 55.27% | 57.48% | 57.34% | 57.37% | - | 0.358 |
| *Bacteroides zoogleoformans* GUS | 61.83% | - | 63.66% | 66.02% | 63.41% | 62.00% | 57.31% | - |

**Table S3.**
**Demographic and clinical characteristics of study participants.**

| **Demographic and Clinical Characteristics of Study Participants (*N*=23)** | | |
|---|---|---|
| Age (mean years ± SD) | 44 ± 17 | |
| | *N* | %*N* |
| Gender | | |
| Female | 15 | 65.2 |
| Male | 8 | 34.8 |
| Ethnicity | | |
| African American | 6 | 26.1 |
| Asian | 1 | 4.3 |
| Caucasian | 16 | 69.6 |
| Periodontitis | | |
| Stage I | 3 | 13 |
| Stage II | 12 | 52.2 |
| Stage III | 8 | 34.8 |

**Table S4.**
**Multilevel models predicting GUS activity inhibition by UNC4917 from GCF samples.**

| | PERIODONTAL PROBING DEPTH (PPD) | | | | PERIODONTITIS CLASSIFICATION (STAGE I, II, or III) | | | |
|---|---|---|---|---|---|---|---|---|
| | Main effects model | | Interaction model | | Main effects model | | Interaction model | |
| | b | 95% CI | b | 95% CI | b | 95% CI | b | 95% CI |
| Intercept | -0.12 | -0.58, 0.33 | -0.62* | -1.18, -0.06 | 0.34** | 0.26, 0.42 | 0.25** | 0.20, 0.30 |
| PPD<br>*3-6 mm range* | 0.24** | 0.13, 0.36 | 0.37** | 0.22, 0.51 | - | - | - | - |
| UNC4917 present<br>*0=no, 1=yes* | -0.24** | -0.35, -0.13 | 0.75* | 0.06, 1.44 | -0.24** | -0.35, -0.13 | -0.06* | -0.10, -0.01 |
| Periodontitis Classification | | | | | | | | |
| Stage I | - | - | - | - | - | - | - | - |
| Stage II | - | - | - | - | 0.29** | 0.20, 0.38 | 0.30** | 0.20, 0.40 |
| Stage III | - | - | - | - | 1.07** | 0.91, 1.24 | 1.32** | 1.10, 1.54 |
| Interactions | | | | | | | | |
| UNC4917 x PPD | - | - | -0.24* | -0.44, -0.05 | - | - | - | - |
| UNC4917 x Periodontitis Classification | | | | | | | | |
| UNC4917 x Stage I (ref) | - | - | - | - | - | - | - | - |
| UNC4917 x Stage II | - | - | - | - | - | - | -0.03 | -0.11, 0.05 |
| UNC4917 x Stage III | - | - | - | - | - | - | -0.49** | -0.74, -0.23 |

Note: ***P*<0.001. * *P*<0.05

**Data S1. (separate file)**
**Protein sequences of identified GUS proteins from the eHOMD.**
